# Screening of gene function in cell intoxication by CNF1 links Sec61 translocon to Rac1 GTPase activity

**DOI:** 10.1128/mbio.02585-24

**Published:** 2025-10-06

**Authors:** Eléa Paillares, Nathalie Deboosere, Stéphane Descorps-Declere, Maud Marechal, Daniel Gillet, Caroline Demangel, Amel Mettouchi, Priscille Brodin, Emmanuel Lemichez

**Affiliations:** 1 Département de Microbiologie, Unité des Toxines Bactériennes, Institut Pasteur, CNRS UMR6047, INSERM U1306, Université Paris Cité 27058, Paris, France; 2 Univ. Lille, CNRS UMR 9017, INSERM U 1019, Institut Pasteur de Lille, US 41-UAR 2014-PLBS, CIIL-Center for Immunity and Infection of Lille 165209, Lille, France; 3 Institut Pasteur, Université Paris Cité, Bioinformatics and Biostatistics Hub 27058, Paris, France; 4 Département Médicaments et Technologies pour la Santé, Université Paris Saclay, CEA, INRAE, SIMoS 27048, Gif-sur-Yvette, France; 5 Institut Pasteur, Université Paris Cité, INSERM U1224, Immunobiology and Therapy Unit 27058, Paris, France; Yale University School of Medicine, New Haven, Connecticut, USA

**Keywords:** CNF1 toxin, Rac1, Rho GTPases, HACE1, Sec61 translocon, mycolactone

## Abstract

The CNF1 toxin from extraintestinal pathogenic *Escherichia coli* (ExPEC) deamidates glutamine 61 of Rac1 small GTPase, as well as its equivalents in RhoA and Cdc42 into glutamic acid. This post-translational modification of Rho proteins abrogates the hydrolysis of GTP into GDP, thereby enhancing signal transduction. Meanwhile, the sustained GTP-loading of Rac1 Q61E sensitizes it to ubiquitin-mediated proteasomal degradation catalyzed by the HACE1 E3 ligase rate-limiting factor, leading to a cellular depletion of Rac1 over time. We report data from a quantitative genome-wide screen of siRNAs inhibiting CNF1-mediated cellular depletion of Rac1 in primary human cells. As best hits, we identified a group of three siRNAs targeting the Sec61A1 subunit of the Sec61 translocon, as well as HACE1 and the Lu/BCAM host cell receptor of CNF1. We extend these findings by identifying a group of siRNAs targeting genes involved in ER and Golgi homeostasis and trafficking. Functional studies showed that both chemical and genetic inhibition of Sec61A1 dampens GTP-loading and membrane association of Rac1 in CNF1-intoxicated cells, while the proper deamidation of RhoA provides a control of CNF1 cytosolic action. Finally, we extend these findings by showing that inhibition of *N*-glycosylation of neo-synthesized proteins in the ER abrogates Rac1 GTP-loading in CNF1-treated cells. Collectively, these data point to a control of Rac1 signaling operated by protein biosynthesis and *N*-glycosylation in the ER.

**IMPORTANCE:**

The remarkable evolutionary convergence of bacterial effectors from pathogens toward the host small GTPase Rac1, the master regulator of the actin cytoskeleton, confers to these microbes an enhanced capacity to invade host cells and tissues. The CNF1 toxin, a colonization factor of the gastrointestinal tract produced by pathogenic strains of *Escherichia coli,* has been instrumental in deciphering the regulation and function of Rac1. By performing a whole-genome screen based on CNF1 action, we establish the key requirement of Sec61 translocon-dependent protein biosynthesis and *N*-glycosylation at the endoplasmic reticulum for proper activation of Rac1 in intoxicated cells. Our data connect the Sec61 translocon and *N*-glycosylation of neo-synthesized proteins at the endoplasmic reticulum in the control of the activity of Rac1 and other Rho GTPases.

## INTRODUCTION

Rac1 small GTPase drives branched actin cytoskeleton polymerization at the interface of membranes downstream of numerous cell signaling pathways controlling cell adhesion, metabolism, division, and fate, as well as gene programs of alarm and defense against pathogen attacks (1–6). Consistent with the central role played by Rac1 in host-pathogen interactions, multiple pathogenic bacteria have evolved key effectors of pathogenicity and colonization targeting this small GTPase (3–6). Rac1 and other Rho proteins, including RhoA and Cdc42, are small GTPases able to switch between a guanosine diphosphate (GDP)-bound inactive form, sequestered in the cytosol in association with a Rho guanine dissociation inhibitor (Rho GDI), and a GTP-bound active form anchored at membranes where they transduce signals (2, 7, 8). How the regulation of Rac1 is integrated within larger signaling networks of cell adaptation to intrinsic stresses largely remains to be defined, considering the master regulatory function of Rac1 in cell fate and defenses against bacterial pathogens.

The CNF1 toxin can enhance the capacities of extraintestinal pathogenic *Escherichia coli* (ExPEC) to colonize the gastrointestinal tract (9). CNF1 binds to the cell surface-exposed Lu/BCAM, and eventually the p67-laminin receptor precursor, to become endocytosed through clathrin- and caveola-independent processes (10–14). The enzymatic part of CNF1 is translocated through vesicular membranes to the cytosol once the toxin reaches the acidic environment of late endosomal compartments (13, 15). CNF1 catalyzes the deamidation of a critical glutamine residue Q61 of Rac1 and Cdc42, or the equivalent Q63 in RhoA, into a glutamic acid (16–18). The deamidation catalyzed by CNF1 onto Rho proteins abrogates their intrinsic and GTPase-activating protein (GAP)-stimulated GTPase activity (16, 17, 19). Consequently, Rho proteins remain locked into a GTP-bound active state, able to transduce signals at the interface of membranes. Whether endogenous GTPase-dead mutants of Rho proteins in CNF1-intoxicated cells undergo a spontaneous loading of guanosine triphosphate or whether the loading of GTP requires the activity of cellular factors remains an open question.

Remarkably, CNF1, by converting endogenous Rho proteins into dominant positive mutant forms, exacerbates a cryptic regulation of Rho protein stability that involves the ubiquitin-proteasomal system (UPS) (19). The degradative polyubiquitination of the GTP-bound form of Rac1 is catalyzed by the HECT-domain and ankyrin repeat-containing E3 ligase 1 (HACE1) (20–22). The activation and proteasomal degradation of Rac1 by the UPS contribute to the invasion of cells and tissues by ExPEC, where beta-1 integrin mechano-activation and signaling are hijacked by bacterial type I pili tipped with FimH (9, 23). Although several cellular targets of HACE1 have been identified, the control exerted by HACE1 on Rac1 is essential in the regulation of ROS production by NADPH oxidase, cyclin-D1 expression, and the tumor suppressor function of HACE1 (21, 24–26). HACE1 accumulates at the edge of membrane ruffles triggered by the ectopic expression of active mutants of Rac1 or in cells intoxicated by CNF1 (20, 27). Moreover, HACE1 displays a Rab1-dependent localization in Golgi stacks, where its E3 ligase activity controls the monoubiquitination of syntaxin-5 and post-mitotic fusion of membranes for Golgi reassembly (28, 29).

A few studies have started to pinpoint connections between Rac1 signaling and the secretory pathway. Notably, Rac1 interacts with the small GTPase Sar1 (secretion-associated Ras-superfamily gene 1) to enhance the formation of endoplasmic reticulum exit sites (ERES), which ensure a supply of lipids to the plasma membrane when under mechanical strains (30). Rac1 and its exchange factor beta-PIX also regulate the recruitment of Arp2/3 to the trans-Golgi network (TGN), where it drives the actin-dependent formation of clathrin-AP1-coated carrier vesicles on the way to the endocytic pathway (31, 32). Finally, it has been reported that expression of the ER-located inositol-requiring transmembrane kinase/endoribonuclease 1 alpha (IRE1 alpha), a sensor and transducer of the unfolded protein response (UPR), controls the levels of active GTP-bound Rac1 in mouse embryonic fibroblasts (MEFs) (33). Much remains to be established on how ER- and Golgi-associated components control Rac1 activity to promote cell homeostasis.

We developed an image-based method to quantify the cellular pool of Rac1, enabling us to assign each siRNA in a pan-human genome screen a score of protection against CNF1-induced depletion of Rac1. This quantitative approach allowed us to pinpoint the critical function of the Sec61A1 subunit of the Sec61 translocon in CNF1-mediated depletion of Rac1, together with a group of genes involved in ER and Golgi trafficking and homeostasis. We report that the blocking effect imposed by knocking down Sec61A1 on the depletion of Rac1 in CNF1-treated cells occurs at the stage of Rac1 activation. The Sec61 translocon forms a ribosome-binding and gated channel, which facilitates the co-translational translocation of nascent proteins in the ER membrane and lumen (34). Remarkably, Sec61 can be specifically inhibited by mycolactone, a polyketide produced by *Mycobacterium ulcerans* (35–37). Recent cryo-electron microscopy data, as well as complementary Sec61A1 mutant-based functional studies, showed that mycolactone docks to Sec61A1 inner channel, thereby locking the Sec translocon in an inactive state (36, 38, 39). As a result, mycolactone inhibits the production of type-I and II transmembrane proteins, as well as the production of most secreted proteins (40, 41). Taking advantage of mycolactone’s defined mode of action, we showed that chemical inhibition of the Sec61 translocon by mycolactone impairs the activation of Rac1 and RhoA in CNF1-treated cells, whereas RhoA is properly deamidated, thereby establishing the intracellular action of the toxin. This suggests that inhibition of the Sec61 translocon uncouples Rho deamidation from the exchange of GDP by GTP, pointing to intrinsic cell-based activation of Rho GTPases in CNF1-intoxicated cells. Together, these data provide evidence that the regulation of Rac1 activity integrates signals from both protein biosynthesis and *N*-glycosylation that emanate from the endoplasmic reticulum.

## RESULTS

### High content screening of siRNAs inhibiting CNF1-induced cellular depletion of Rac1

**Fig 1:**
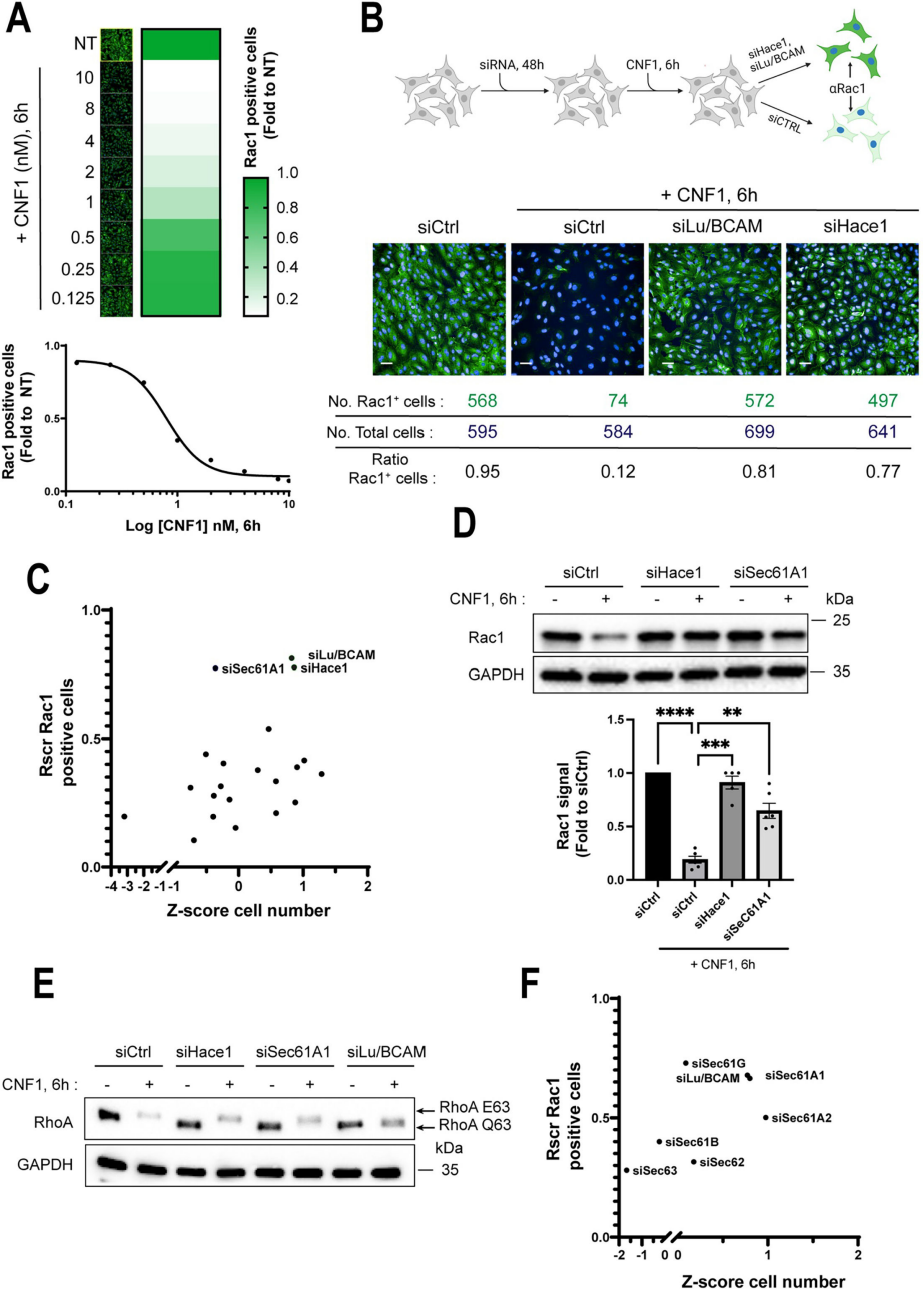
High-throughput screening of siRNA inhibiting CNF1-induced Rac1 depletion. (**A**) Representative confocal images of endogenous Rac1 immunofluorescence signal intensities (left panels, green) in control and CNF1-intoxicated cells, as well as a heat map (right panels) showing the number of Rac1-positive cells expressed as fold to non-treated conditions (NT). HUVECs were treated with the indicated concentrations of CNF1 in nanomolar (nM) for 6 h, and images were acquired on an Opera Phenix automatic confocal microscope. The lower graph shows the percentage of Rac1-positive cells as a function of the log_10_ values of CNF1 concentrations. Dots show mean values of *n* = 3 independent experiments, *n* = 5 replicates. Linear regression shows the linear relationship (*R*^2^ = 0.62) between values of Rac1-positive cells and a range of CNF1 concentrations comprised between 2 and 0.5 nM, with a calculated EC_50_ value of 0.8 nM. (**B**) The upper panel shows the principle of the screen designed to identify siRNAs inhibiting CNF1-induced cellular depletion of Rac1. HUVEC transfected with siCtrl and treated with CNF1 set the minimal value of endogenous Rac1 signal (αRac1). HUVEC was transfected with siLu/BCAM or siHACE1, and then intoxicated with CNF1, resulting in maximal protection values of Rac1 signals. The lower panels show representative confocal images of HUVEC transfected with siCtrl, siLu/BCAM, or siHace1 and treated with CNF1 at 1 nM for 6 h, as well as siCtrl cells left untreated. Confocal images acquired on Opera Phenix at objective 20× show endogenous Rac1 immunofluorescence (green) and nuclei (blue). Scale bar, 50 µm. Values show the number of Rac1-positive cells (No. Rac1+cells), total cells (No. total cells), and the calculated ratio of Rac1+ cells. (**C**) Scatter plot showing the distribution of Rscr values of Rac1-positive cells transfected with 21 reordered siRNAs from the list of top-ranked siRNA (confirmatory screens), as a function of the calculated Z-score of cell number. Cells were treated with CNF1 at 1 nM for 6 h. Each dot corresponds to mean values from *n* = 3 independent experiments, *n* = 4 replicates. (**D**) Immunoblots show endogenous Rac1 signals from cells transfected with siCtrl, siHace1, and siSec61A1 that were left untreated or intoxicated with 1 nM of CNF1 for 6 h. GAPDH shows equal protein loading control. One representative experiment, *n* = 5. The lower graph shows intensities of Rac1 signals normalized to GAPDH and expressed as fold to the siCtrl condition. Data are mean ± SEM. Each dot corresponds to an individual value from *n* = 5 independent experiments. One-way ANOVA with Dunnett’s correction; siCtrl+ CNF1 vs siCtrl, ****P < 0.0001; siCtrl+ CNF1 vs siHace1+ CNF1, ***P = 0.0008; siCtrl+ CNF1 vs siSec61A1 + CNF1, **P = 0.0062. (**E**) Immunoblots show the upper shift that undergoes the deamidated form of RhoA (RhoA Q63E) in CNF1-intoxicated cells, versus wild-type RhoA (RhoA Q63) in Urea-SDS-PAGE condition. The cells were transfected with siCtrl, siSec61A1, siHace1, or siLu/BCAM and left untreated or intoxicated with CNF1 at 1 nM for 6 h. Immunoblot GAPDH shows equal protein loading. One representative experiment, *n* = 3. (**F**) Scatter plot showing the distribution of Rscr values of Rac1-positive cells transfected with six siRNAs targeting structural and regulatory subunits of the Sec61 translocon, as well as siLu/BCAM, as a function of the calculated Z-score of cell number. Cells were treated with CNF1 at 1 nM for 6 h. Each dot corresponds to mean values from *n* = 2 independent experiments, *n* = 6 replicates.

The CNF1 toxin induces a time- and dose-dependent depletion of the GTP-bound active form of Rac1 (14, 19, 20). We aimed to exploit this property of CNF1 to screen for new regulatory components of Rac1. We developed an image-based quantitative method to measure the cellular levels of endogenous Rac1. This method relies on the fluorescent immunolabeling of Rac1, coupled to an automated imaging algorithm that segments and enumerates Rac1-positive cells within the whole cell population (Fig. S1A). The CNF1 toxin was first applied to human umbilical vein endothelial cells (HUVECs) for 6 h in 2-fold serial dilutions from 10 nM to 0.125 nM. The percentage of Rac1-positive cells showed a linear decrease between 0.5 nM and 2 nM of CNF1, R^2^ = 0.62 (Fig. 1A). The above data prompted us to perform the screen with CNF1 at 1 nM, that is, within the dynamic range of endogenous Rac1 signal detection (Fig. 1A). To set up the assay for gene knockdown, the cells were transfected with small interfering RNAs (siRNAs) targeting Lu/BCAM, to block the endocytosis of CNF1, or HACE1, to block the ubiquitin-mediated degradation of Rac1 (Fig. 1B; Fig. S1B). In siLu/BCAM and siHACE1 conditions, we recorded 81% and 77% of Rac1-positive cells treated with CNF1, respectively, whereas the levels of Rac1-positive cells dropped to 12% in the siRNA control (siCtrl) and CNF1 condition (Fig. 1B). Given the robustness of these effects, siHace1 and siCtrl in the absence or presence of CNF1 were chosen as positive and negative controls, respectively. We then screened a genome-wide siRNA library targeting 18,125 human genes. As a quality control, we determined an SSMD value for each plate using internal controls (e.g., cells treated with siCtrl or siHace1 in the absence or presence of CNF1) (Fig. S1C). This allowed us to retain a total of 60 plates (*n* = 384 wells each), encompassing a total of 16,433 siRNAs, for further analysis. Due to the non-random organization of the siRNA library, we calculated a plate-based normalization score, Rscreenorm (Rscr), of Rac1-positive cells using a method previously described (42). Within the ranked siRNAs of the primary screen, we identified siRNAs targeting HACE1 (position 3, Rscr = 0.86) and Lu/BCAM (position 22, Rscr = 0.79), supporting the robustness of the screen ( Table S1). We reordered 21 siRNAs with Rscr scores ≥ 0.79 (including siLu/BCAM) to perform three independent confirmatory screens and ranked them according to the average of their protection scores (Table 1 and Fig. 1C). Remarkably, we identified a group of three best hits encompassing siLu/BCAM and siHace1 together with an siRNA targeting the Sec61A1 subunit of the Sec61 translocon (Rscr > 0.77) (Fig. 1C; Table 1). For the remaining 18 siRNAs, we measured lower Rscr scores comprised between 0.36 and 0.54 (Fig. 1C; Table 1). Immunoblotting, as an alternative method of analysis, confirmed the high level of protection conferred by siSec61A1 against CNF1-induced depletion of Rac1 (Fig. 1D). The decrease in Sec61A1 mRNA in siSec61A1-knockdown cells was confirmed by qPCR (Fig. S1D). To rule out a potential interference of siSec61A1 in the CNF1 intoxication process, we took advantage of the unique property of the deamidated form of RhoA (RhoA Q63E), compared with wild-type RhoA among Rho proteins, to exhibit an electrophoretic mobility shift on urea-SDS-PAGE (43). This analytical method enables quantitative monitoring of the intracellular activity of the CNF1 toxin. HUVECs were treated with siCtrl, siLu/BCAM, siHace1, or siSec61A1 before the addition of CNF1 at 1 nM for 6 h. In siLu/BCAM-treated cells, which are no longer susceptible to CNF1, we did not detect the electrophoretic mobility shift of RhoA (Fig. 1E). In contrast, we observed the shifted form of RhoA in cells co-treated with siSec61A1 and CNF1, thereby indicating productive intoxication (Fig. 1E). Finally, we went on to confirm the protection conferred by siRNAs targeting core and accessory components of the Sec61 translocon including Sec61A, Sec61B, Sec61G, Sec62, and Sec63, against CNF1-induced depletion of Rac1, yielding Rscr values between 0.28 and 0.72 (Fig. 1F). This genome-wide quantitative screen provides a list of gene candidates involved in CNF1-induced cellular depletion of Rac1, also assigning a key function to Sec61A1 channel subunit of the Sec61 translocon.

**TABLE 1:** List of 21 siRNA-targeted genes ranked according to data of confirmatory screens^*a*^

No.	Gene	Screen name	Ref_Seq	Rscreenorm confirmation	Shift RhoA
1	Lu/BCAM	LU/BCAM	NM_001013257	0.81	N
2	HACE1	HACE1	NM_020771	0.78	Y
3	SEC61A1	SEC61A1	NM_013336	0.77	Y
4	USO1	VDP	NM_003715	0.54	NA
5	VSIG8	LOC391123	NM_001013661	0.44	Y
6	RGS7BP	LOC401190	NM_001029875	0.42	Y
7	SWSAP1	FLJ35119	NM_175871	0.40	Y
8	SLF1	ANKRD32	NM_032290	0.39	Y
9	SH3D19	SH3D19	NM_001009555	0.38	NA
10	SLC35E1	SLC35E1	NM_024881	0.36	NA
11	CYB561A3	CYB561A3	NM_153611	0.33	NA
12	TRUB2	TRUB2	NM_015679	0.32	NA
13	TBC1D22A	C22ORF4	NM_014346	0.31	NA
14	PTCD1	PTCD1	NM_015545	0.28	NA
15	TMEM43	TMEM43	NM_024334	0.26	NA
16	CFAP77	FLJ46082	NM_207417	0.25	NA
17	HNRNPUL2	HNRPUL2	NM_001079559	0.21	NA
18	ZNF598	ZNF598	NM_178167	0.20	NA
19	HNRNPA2B1	HNRPA2B1	NM_002137	0.20	NA
20	OR2T6	OR2T6	NM_001005471	0.15	NA
21	LIN9	LIN9	NM_173083	0.10	NA

^*a*^ List of the top-21 siRNAs inhibiting CNF1-induced Rac1 cellular depletion ranked according to mean values of Rscr defined in *n *= 3 independent confirmatory experiments with triplicates (confirmed Rscr). From left to the right: gene number, gene name (Ref_seq), confirmed Rscr, deamidated RhoA detected in CNF1-treated cells (Y for yes; N for No and NA for not assessed). SiRNA targeted gene names according to GeneCards: Human Genes (https://www.genecards.org) are as follows: Lu/BCAM: basal cell adhesion molecule (Lutheran blood group); Sec61A1: Sec61 translocon subunit alpha 1; HACE1: HECT domain and ankyrin repeat containing E3 ubiquitin protein ligase 1; RGS7BP: regulator of G protein signaling 7 binding protein; SWAP1: SWIM-type zinc finger 7 associated protein 1; VSIG8: V-Set and immunoglobulin domain containing 8; SLF1: SMC5-SMC6 complex localization factor 1; SH3D19: SH3 domain containing 19; CYB561A3: cytochrome B561 family member A3; TBC1D22A: TBC1 domain family member 22A; PTCD1: pentatricopeptide repeat domain 1; TRUB2: TruB pseudouridine synthase family member 2; TMEM43: transmembrane protein 43; HNRNPUL2: heterogeneous nuclear ribonucleoprotein U like 2; HNRNPA2B1: heterogeneous nuclear ribonucleoproteins A2/B1; CFAP77: cilia and flagella associated protein 77; ZNF598: zinc finger protein 598; LIN9: Lin-9 DREAM MuvB core complex component; OR2T6: olfactory receptor 2T6.

### Chemical inhibition of the Sec61 translocon abrogates CNF1-mediated depletion of Rac1

**Fig 2:**
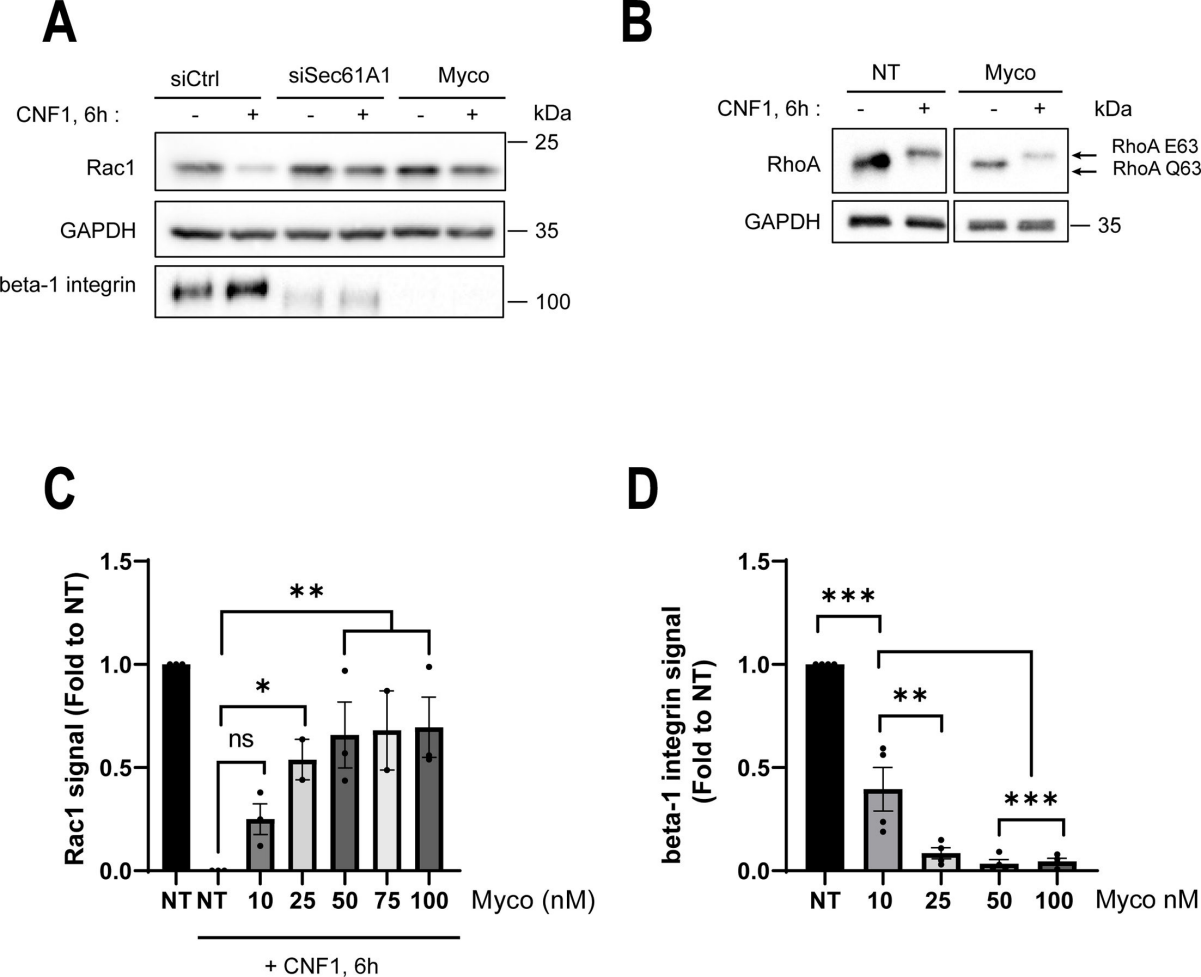
Active Sec61 translocon is required for CNF1-induced Rac1 depletion. (**A**) Immunoblots show endogenous Rac1 immunofluorescence signal intensities from cells treated with siCtrl, siSec61A1, or treated with mycolactone (Myco) at 100 nM for 24 h, and prior to treatment with 1 nM of CNF1 for 6 h. Immunoblot GAPDH shows equal protein loading. An immunoblot anti-beta-1 integrin was performed to control the inhibition of protein biosynthesis in the ER. One representative experiment, *n* = 3. (**B**) Immunoblots show the electromobility shift of RhoA Q63E versus RhoA Q63 in cells treated with mycolactone at 100 nM for 24 h (Myco) prior to treatment with 1 nM CNF1 for 6 h. Immunoblot GAPDH shows equal protein loading. One representative experiment, *n* = 3. (**C**) The graph shows intensities of Rac1 signals normalized to GAPDH and expressed as fold to the non-treated (NT) condition. Cells were treated for 24 h with mycolactone (Myco) at the indicated concentrations prior to treatment with 1 nM of CNF1 for 6 h. Bars show mean ± SEM. Each dot corresponds to an individual value from *n* = 3 independent experiments. One-way ANOVA with Dunnett’s correction; NT + CNF1 vs Myco 10 nM + CNF1, ns; NT + CNF1 vs Myco 25 nM + CNF1, *P = 0.035; NT + CNF1 vs Myco 50 nM + CNF1, **P = 0.044; NT + CNF1 vs Myco 75 nM + CNF1, **P = 0.079; NT + CNF1 vs Myco 100 nM + CNF1, **P = 0.029. (**D**) The graph shows intensities of beta-1 integrin signals normalized to GAPDH and expressed as fold to the non-treated (NT) condition. Cells were treated for 24 h with mycolactone (Myco) at indicated concentrations prior to treatment with CNF1 at 1 nM for 6 h. Bars show mean ± SEM. Each dot corresponds to an individual value from *n* = 4 independent experiments. One-way ANOVA with Dunnett’s correction; Myco 10 vs NT, ***P < 0.001; Myco 10 vs Myco 25, **P = 0.0019; Myco 10 vs Myco 50, ***P = 0.0005; Myco 10 vs Myco 100, ***P = 0.0007.

As an orthogonal approach, we tested whether chemical inhibition of the Sec61 translocon with mycolactone had an inhibitory effect on the depletion of Rac1 in CNF1-treated cells. Cells were incubated with 100 nM mycolactone for 24 h before the addition of the toxin at 1 nM for 6 h. In cells co-treated with mycolactone and CNF1, the level of endogenous Rac1 remained high as opposed to the control condition (Fig. 2A). The proper inhibition of Sec61 translocon activity was monitored by immunoblotting the beta-1 integrin transmembrane protein (Fig. 2A). In parallel, we verified that the treatment of cells with mycolactone at 100 nM did not affect the capacity of CNF1 to deamidate RhoA (Fig. 2B). By conducting dose-dependent experiments, we recorded a significant stabilization of Rac1 signal with concentrations of mycolactone of 25 nM, and above, which efficiently decreased the cellular pool of beta-1 integrin (Fig. 2C and D). Together, these findings extend data from the genetic screen, highlighting the requirement of a functional Sec61 translocon for the proper depletion of Rac1 in CNF1-treated cells.

### Functional Sec61 translocon enables CNF1-mediated association of Rac1 to membranes

**Fig 3:**
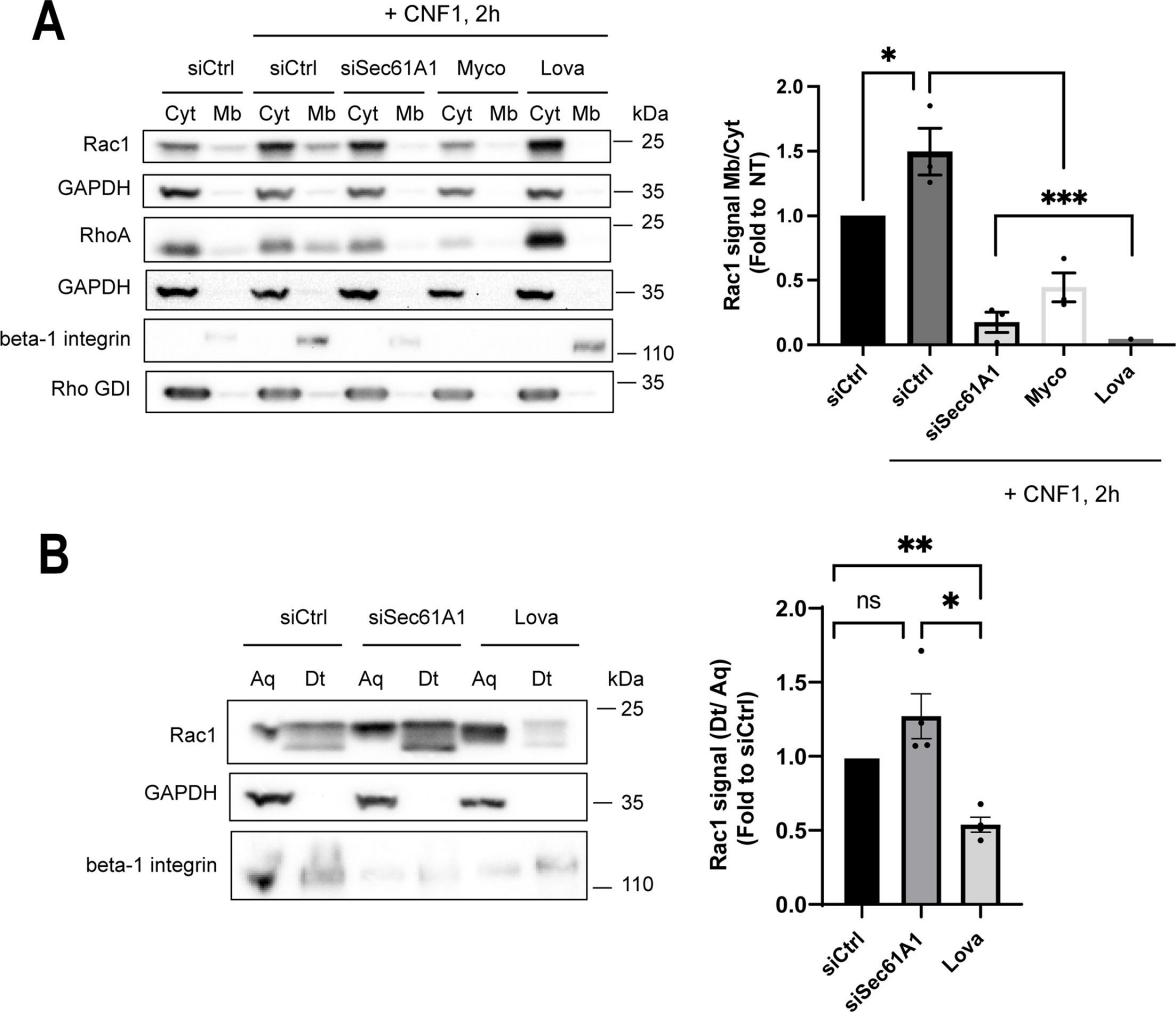
Functional Sec61 translocon allows CNF1-induced translocation of Rac1 to membranes. (**A**) Immunoblots show the relative distribution of Rac1 and RhoA between cytosol (Cyt) and membrane (Mb) fractions in HUVEC intoxicated with CNF1. Cells were pre-treated with siCtrl, siSec61A1, 100 nM mycolactone (Myco), or 15 µM lovastatin (Lova) 24 h prior to intoxication with CNF1 at 1 nM for 2 h. Immunoblots anti-Rho GDI or anti-GAPDH show controls of membrane fractionation and equal loading of cytosolic proteins. Immunoblot anti-beta-1 integrin shows control of the inhibition of protein biosynthesis in the ER. Representative immunoblots of *n* = 3 independent experiments. Rac1 immunosignals were quantified and are expressed as the signal ratio between membrane and cytosol, together with membrane fractions normalized to NT (graph). Bars show means ± SEM. Each dot corresponds to an individual value from *n* = 3 independent experiments. One-way ANOVA with Dunnett’s correction, siCtrl vs siCtrl + CNF1, *P = 0.4966; siCtrl + CNF1 vs siSec61A1 + CNF1, ***P = 0.0001; siCtrl + CNF1 vs siCtrl + Myco + CNF1, ***P = 0.0006; siCtrl + CNF1 vs siCtrl + Lova + CNF1, ***P = 0.0007. (**B**) Immunoblots show the distribution of Rac1 between aqueous (Aq) and detergent (Dt) phases after Triton X-114 nonionic detergent phase separation. HUVECs were transfected with siSec61A1 or siCtrl for 24 h prior to the extraction of prenylated-Rac1 in the Triton X-114 phase. As a control, siCtrl-transfected cells were treated with 15 µM of lovastatin for 24 h (Lova). Immunoblot anti-GAPDH shows control of phase separation. Immunoblot anti-beta-1 integrin shows control of Sec61 translocon inhibition. One representative experiment, *n* = 4. Rac1 signals were quantified and expressed as signal ratio between the detergent and aqueous phases, normalized to the siCtrl condition (graph). Bars show means ± SEM. Each dot corresponds to an individual value from *n* = 4 independent experiments. One-way ANOVA with Dunnett’s correction, siCtrl vs siSec61A1, ns; siCtrl vs siCtrl + Lova, **P = 0.0058; siSec61A1 vs siCtrl + Lova, *P = 0.0452.

We investigated whether Sec61A1 plays a role in Rac1 recruitment to cellular membranes upon CNF1 stimulation. Indeed, in cells co-treated with siSec61A1 and CNF1, we observed that Rac1 remained diffusely distributed in the cytosol (Fig. S2). This prompted us to fractionate the cellular membranes from the cytosol and assess the relative distribution of Rac1 in membrane and cytosolic fractions. The efficient enrichment of membranes from cytosolic components was controlled by immunoblotting glyceraldehyde-3-phosphate dehydrogenase (GAPDH), a cytosolic protein, as well as the Rho GDI factor that sequesters the GDP-bound form of Rho proteins in the cytosol (Fig. 3A). Cells were treated with CNF1 at 1 nM for 2 h to promote the activation of Rac1 over its degradation and therefore raise the level of membrane-associated Rac1 (Fig. 3A). As a control, we treated cells with lovastatin, an inhibitor of HMG-CoA reductase, the rate-limiting enzyme in the mevalonate pathway. This treatment blocks the prenylation of Rho proteins, preventing their association with cellular membranes. Consistently, in the condition of lovastatin treatment, we no longer observed a presence of Rac1 in the membrane fraction of cells intoxicated by CNF1 (Fig. 3A). Finally, when CNF1-intoxicated cells were co-treated with siSec61A1 or mycolactone, we observed an inhibition of the targeting of Rac1, as well as RhoA, to membranes (Fig. 3A). We next tested whether inhibition of the Sec61 translocon might interfere with the prenylation of Rac1, considering that HMG-CoA reductase is an integral membrane protein of the endoplasmic reticulum (44). We proceeded with the extraction of the prenylated form of Rac1 from total cell extracts by phase separation with Triton X-114, as previously described (45). Although lovastatin treatment reduced the levels of Rac1 in the detergent fraction, we recorded similar signal intensities of Rac1 between aqueous and detergent fractions in siCtrl and siSec61A1 conditions (Fig. 3B). We concluded that siSec61A1 had no detectable impact on the efficiency of Rac1 prenylation. Altogether, these results establish that a functional Sec61 translocon is required for the targeting of prenylated Rac1 to membranes in CNF1-treated cells.

### Functional Sec61 translocon enables GTP-loading of Rac1, RhoA, and Cdc42 in CNF1-intoxicated cells

**Fig 4:**
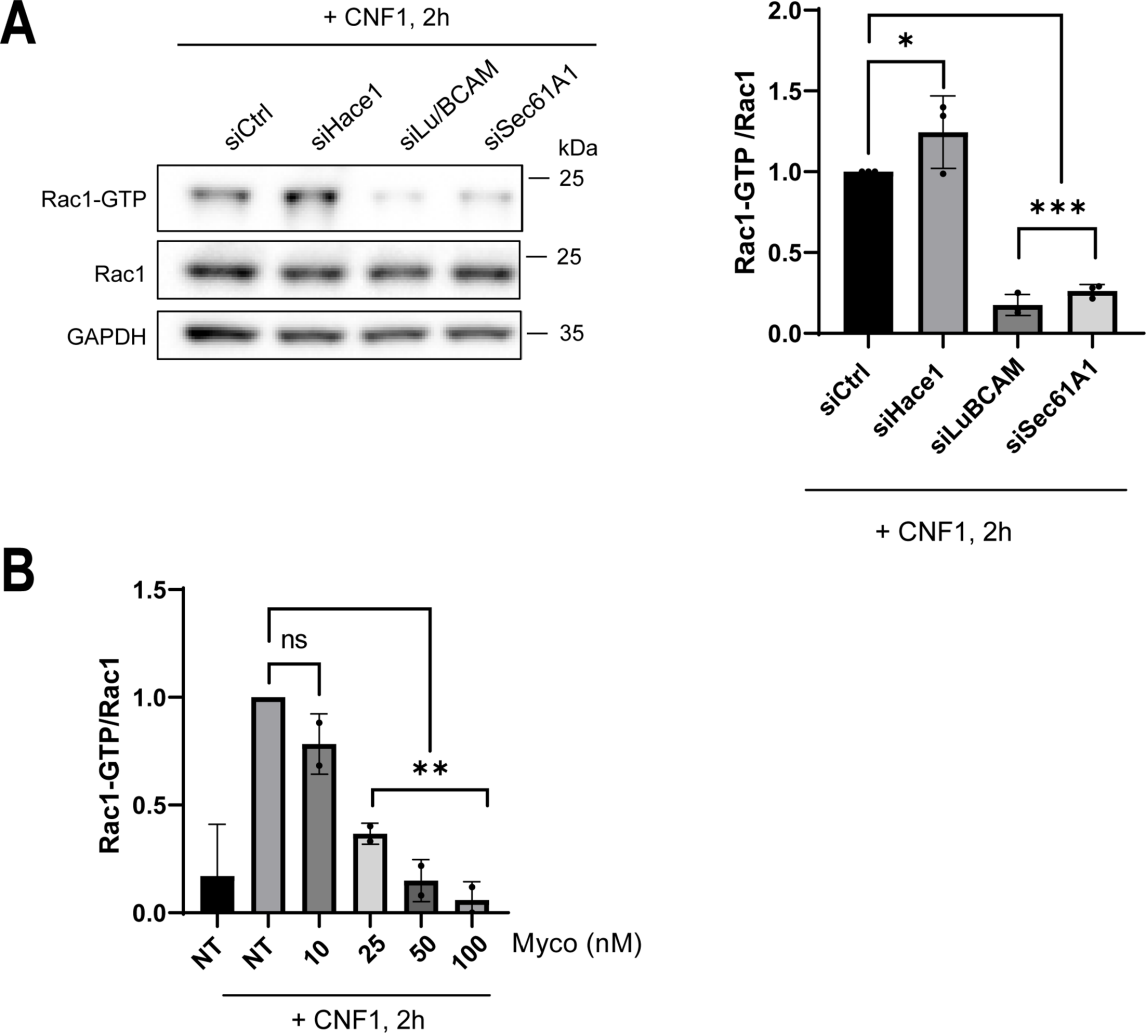
Active Sec61 translocon allows CNF1-mediated activation of Rac1. (**A**) Representative immunoblots show levels of GTP-bound Rac1 (Rac1-GTP) in cells transfected with siCtrl, siHace1, siLu/BCAM, or siSec61A1 prior to intoxication with CNF1 at 1 nM for 2 h. Levels of GTP-bound Rac1 were assessed by GST-PAK pulldown. Immunoblots of anti-GAPDH and anti-Rac1 on total cell lysates show equal protein amounts engaged in the GST-PAK pulldown. Graph: Rac1 immunosignals were quantified and are expressed as the ratio of GTP-bound Rac1 normalized to the total Rac1 signal. Bars show means ± SEM. Each dot corresponds to a value from *n* = 3 independent experiments. One-way ANOVA with Dunnett’s correction, siCtrl vs siLu/BCAM, ***P = 0.0041; siCtrl vs siSec61A1, ***P = 0.0019. (**B**) Graph shows the variation of GTP-bound Rac1 (Rac1-GTP) levels normalized to Rac1 total level (Rac1) in cells left untreated (NT) or treated for 24 hours with mycolactone (Myco) at indicated concentrations together with CNF1 at 1 nM. Data are expressed as fold variations compared with cells treated with CNF1. Levels of Rac1-GTP were assessed by GST-PAK pulldown and anti-Rac1 immunoblotting. Bars show means ± SEM. Each dot corresponds to a value from *n* = 2 independent experiments. One-way ANOVA with Dunnett’s correction, NT vs Myco 10, ns; NT vs Myco 25, **P = 0.0089; NT vs Myco 50, **P = 0.002; NT vs Myco 100, **P = 0.0012.

We then assessed whether a functional Sec61 translocon is required for GTP-loading of Rho proteins in cells treated with CNF1. To this aim, we first measured the cellular levels of GTP-bound Rac1 and Cdc42 by pulldown using the p21-PAK effector binding (CRIB) domain fused to GST as bait (46). We first knocked down Hace1 and Lu/BCAM to establish the maximum and minimum levels of activation of Rac1 in cells intoxicated with CNF1 at 1 nM for 2 h, respectively (Fig. 4A). Interestingly, we observed a sharp decrease in the cellular pool of GTP-bound Rac1 when cells were treated with siSec61A1 prior to CNF1 intoxication (Fig. 4A). In these experimental conditions, we also measured a decrease in the cellular pool of GTP-bound Cdc42 (Fig. S3A). Complementary to these findings, we recorded a dose-dependent decrease of GTP-bound Rac1 in CNF1-intoxicated cells treated simultaneously with different concentrations of mycolactone, thereby establishing an EC_50_ value around 25 nM (Fig. 4B). As a control, we recorded a massive inhibitory effect of mycolactone on the expression of beta-1 integrin at a concentration of 25 nM and above (Fig. S3B). We extended these findings by showing that the inhibition of the Sec61 translocon with siSec61A1 or mycolactone in CNF1-treated cells decreased the pool of GTP-bound RhoA (Fig. S3C). Finally, we verified that mycolactone treatment inhibited CNF1-induced Rac1 activation in MCF12A cells, confirming the requirement of Sec61 in a different cell type (Fig. S3D). Together, these data establish that Sec61 translocon activity is essential for increasing the cellular levels of GTP-bound Rac1 in CNF1-treated cells.

### Inhibition of protein *N***-**glycosylation abrogates the CNF1-mediated activation of Rac1

**Fig 5:**
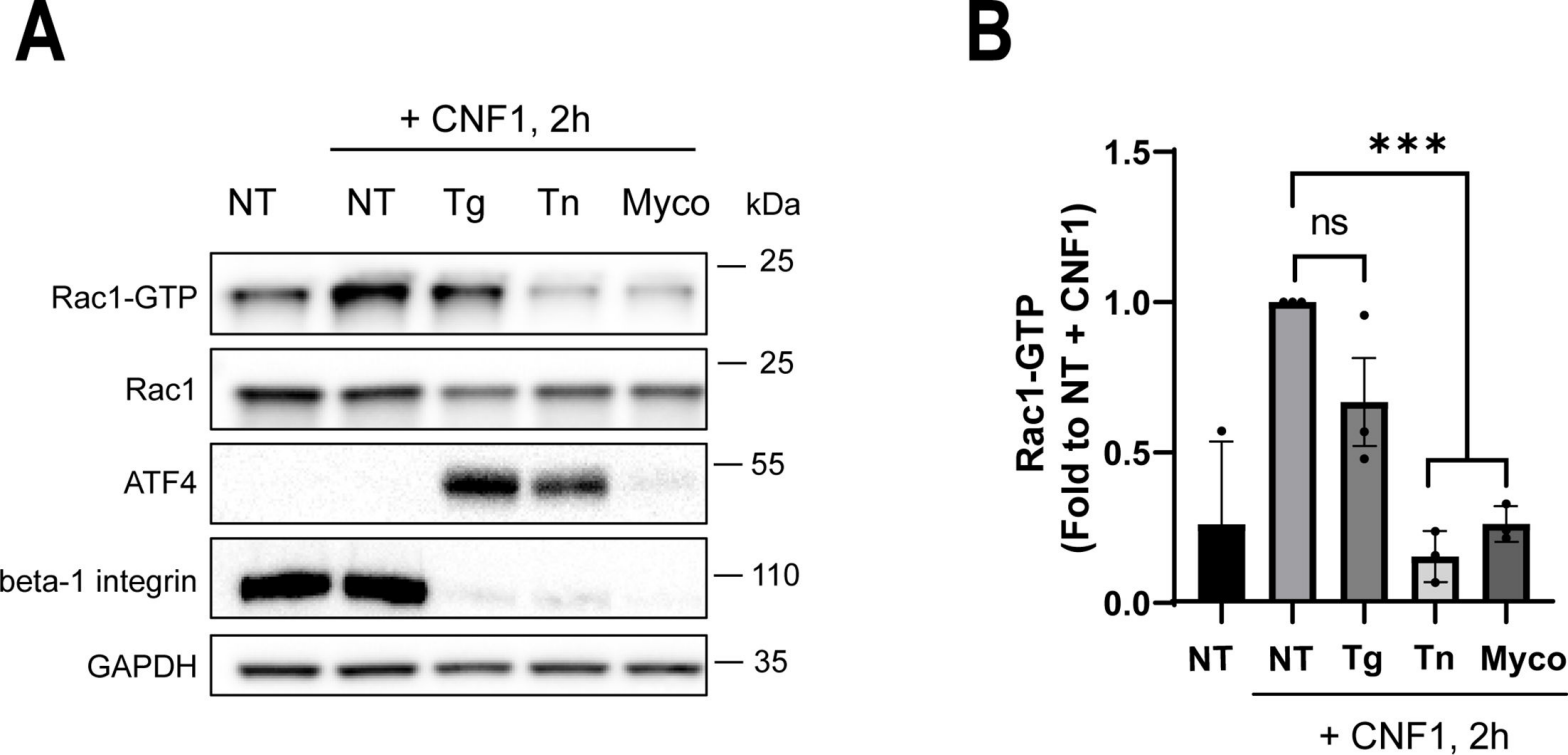
ER stress induced by tunicamycin blocks CNF1-mediated activation of Rac1. (**A**) Representative immunoblots show GTP-bound Rac1 (Rac1-GTP) levels in cells treated with ER stressors. HUVECs were treated for 24 h with 2 µM thapsigargin (Tg), 2 µM tunicamycin (Tn), or 100 nM mycolactone (Myco) prior to the addition of CNF1 at 1 nM for 2 h. The fraction of GTP-bound Rac1 was purified by GST-PAK pulldown. Immunoblots anti-GAPDH and anti-Rac1 show equal protein amounts engaged in GST-PAK pulldowns. Immunoblots anti-beta-1 integrin and anti-ATF4 show Sec61 translocon inhibition and induction of PERK-dependent ER stress signaling, respectively. Representative immunoblots, *n* = 3 independent experiments. (**B**) Graph shows levels of GTP-bound Rac1 normalized to GAPDH and expressed as fold inhibition of Rac1 GTP compared with untreated cells. Bars show means ± SEM. Each dot corresponds to an individual value from *n* = 3 independent experiments. One-way ANOVA with Dunnett’s correction, NT vs Tg, ns; NT vs Tn ***P = 0.0002; NT vs Myco ***P = 0.0006.

The ER is a defined compartment that fulfills critical physiological functions as a site of membrane and secretory protein synthesis, and post-translational modifications, also including quality control mechanisms that ensure the exit of properly folded proteins. The unfolded protein response (UPR) sensors monitor the folding status of neosynthesized proteins in the ER to adjust the flux of protein synthesis and the fate of cells. The UPR encompasses three established pathways, for example, the protein kinase RNA-like ER kinase (PERK)/ATF4, the IRE1alpha/XBP1, and ATF6 (47). The ER-resident transmembrane proteins PERK, IRE1alpha, and ATF6 act as sensors of protein unfolding and transducers via the post-translational modifications of cytosolic proteins or induction of genetic programs in the nucleus. Mycolactone is a known ER stressor that launches adaptive cell reactions to UPR involving PERK and IRE1alpha (48–50). This prompted us to test whether different ER stressors might inhibit the activation of Rac1 in CNF1-treated cells. To this end, cells were treated with thapsigargin (Tg) or tunicamycin (Tn) that trigger ER stresses via inhibition of endoplasmic reticulum Ca^2+^-ATPase SERCAs or the inhibition of *N*-linked glycosylation of neosynthesized proteins in the ER, respectively (51, 52). HUVECs were treated for 24 h with mycolactone, Tn, or Tg, followed by intoxication with CNF1 at 1 nM for 2 h to raise the cellular pool of GTP-bound Rac1. The induction of ER stress responses was monitored by recording the expression of ATF4 (Fig. 5A). In parallel, we observed a decrease in the pool of GTP-bound Rac1 in CNF1-intoxicated cells co-treated with Tn or mycolactone (Fig. 5B). Conversely, the ER stressor Tg had no impact on the pool of GTP-bound Rac1 in CNF1-treated cells, whereas it induced a strong expression of ATF4 (Fig. 5A and B). We verified that CNF1 induced a proper deamidation of RhoA in cells treated with Tg, Tn, and mycolactone (Fig. S4). Taken collectively, our data point to the importance of *N*-glycosylation of the flux of neosynthesized protein in controlling the pool of GTP-bound Rac1 in CNF1-treated cells.

### Crosstalk between Rac1 Q61E depletion and regulators of ER and Golgi vesicular trafficking

The above data prompted us to search for a possible interference of components of the ER and Golgi secretory pathway in CNF1-mediated depletion of Rac1. To this aim, we performed a second-pass reanalysis of the best-ranked 260 siRNA hits of the primary screen (Rscr > 0.6) for their annotated function in vesicular trafficking, including components of the ER and Golgi secretory pathway (Materials and Methods). This ontology-based analysis led to selecting 22 siRNAs. These siRNAs were reordered and ranked according to the average of Rscr values defined in three independent experiments (Table 2). In total, 77.2% of the siRNA (*n* = 17) passed the confirmatory tests with recorded values of Rscr above 0.1 (Table 2). Note that assigned values of protection defined in this secondary screen further confirmed the major inhibitory effect triggered by siSec61A1 on the CNF1-induced cellular depletion of Rac1. The first seven siRNAs showed no impact on CNF1-induced deamidation of RhoA, pointing to an absence of effect on CNF1 action. Consistent with our hypothesis of an interplay between the flux of protein biosynthesis in the ER and Rac1 signaling, we found five siRNAs among the first seven hits that displayed a function assigned to ER and Golgi trafficking and homeostasis (Table 2). In conclusion, we identified siRNAs targeting genes having a function assigned to ER and Golgi homeostasis and trafficking for their inhibitory effect on CNF1-induced depletion of Rac1.

**TABLE 2:** List of 22 siRNA-targeted genes ranked according to data of confirmatory screens^*a*^

No.	Gene	Ref_Seq	Rscr confirmation	Shift RhoA
1	**SEC61A1**	NM_013336	0.78	Y
2	**PITPNB**	NM_012399	0.60	Y
3	SNX4	NM_003794	0.58	Y
4	**TMED9**	NM_017510	0.50	Y
5	SEL1L	NM_005065	0.48	Y
6	GBF1	NM_004193	0.45	Y
7	RAB11A	NM_004663	0.42	Y
8	**USO1/VDP**	NM_003715	0.41	NA
9	COMMD8	NM_017845	0.35	NA
10	**SLC35E1**	NM_024881	0.30	Y
11	**RAB41**	NM_001032726	0.29	NA
12	HIPK2	NM_022740	0.27	NA
13	COMMD2	NM_016094	0.21	NA
14	CYB561A3	NM_153611	0.16	NA
15	COMMD5	NM_001081004	0.16	NA
16	PODXL	NM_005397	0.16	NA
17	FAM109A	NM_144671	0.13	NA
18	EXOC7	NM_015219	0.08	NA
19	GNL3	NM_014366	0.06	NA
20	**AGAP1**	NM_014914	0.01	NA
21	**ARAP3**	NM_022481	0.00	NA
22	HGS	NM_004712	−0.11	NA

^*a*^ List of the siRNAs annotated for their function in vesicular trafficking on neXtProt among the top-260 siRNAs of the primary screen and ranked according to mean values of Rscr defined in *n *= 3 independent experiments performed in triplicate (confirmed Rscr). From left to the right: gene number, gene name (Ref_seq), confirmed Rscr, deamidated RhoA detected in CNF1-treated cells (Y) or not (N) or not assessed (NA). The siRNA-targeted genes with a function in ER and Golgi homeostasis and transport are indicated in bold. The siRNA-targeted gene names according to GeneCards: Human Genes (https://www.genecards.org) are as follows: PITPNB: phosphatidylinositol transfer protein beta isoform; SNX4: sorting nexin-4; TMED9: transmembrane Emp24 domain-containing protein 9; SEL1L: the SEL1L adaptor subunit of SYVN1 ubiquitin ligase; GBF1: Golgi-specific brefeldin A-resistance guanine nucleotide exchange factor 1; Rab11A: Ras-related protein Rab 11A; USO1: USO1 vesicle transport homologue; COMMD8: COMM domain containing 8; SELC35E1: solute carrier family 35 member E1; Rab41: Ras-related protein Rab-41; HIPK2; homeodomain-interacting protein kinase 2; TP53: tumor suppressor P53; COMMD2: COMM domain-containing 2; CYB561A3: cytochrome B561 family member A3; COMMD5: COMM domain-containing 5; PODXL: podocalyxin-like protein 1; FAM109A: PH domain containing endocytic trafficking adaptor 1; EXOC7: exocyst complex component Exo70; GNL3: guanine nucleotide binding protein-Like 3; AGAP1: Arf-GAP with GTPase, ANK repeats and PH domain-containing protein 1; ARAP3: Arf-GAP with Rho-GAP domain, ANK repeats and PH domain-containing protein 3; HGS: human growth factor-regulated tyrosine kinase substrate.

## DISCUSSION

By conducting a genome-wide screen, we highlight gene candidates involved in CNF1-induced cellular depletion of Rac1. Most notably, the implementation of a quantitative approach highlights the critical role of the Sec61 translocon in CNF1-mediated activation of Rac1. The importance of the Sec61 translocon machinery as a whole is supported by the recorded inhibitory impact on CNF1-mediated depletion of Rac1 of siRNAs targeting core and regulatory components of the Sec61 translocon. Moreover, we establish with mycolactone the requirement of Sec61 translocon activity on CNF1-mediated depletion of Rac1. We did not identify the precise molecular circuit integrating the activity of Sec61 translocon in Rac1 signaling. Nevertheless, we show that both the knockdown and chemical inhibition of Sec61A1 inhibit the GTP-loading of Rac1 and its targeting to membranes downstream of Rac1 prenylation. Moreover, we report that the activation of Rac1 in CNF1-treated cells requires the proper *N*-glycosylation of neosynthesized proteins consistent with the idea that the GTP-loading of Rac1 is subjected to a control by the flow of protein biosynthesis in the ER. This idea is enforced by our findings of a panel of genes involved in ER/Golgi trafficking and homeostasis that interfere with the CNF1-mediated depletion of Rac1. Collectively, these data open new avenues in studying the crosstalk between the regulation of Rac1 and compartmentalized biosynthesis of integral membrane and secreted proteins at the endoplasmic reticulum.

The CNF1 toxin represents an example of an environmental factor that catalyzes a somatic alteration of endogenous Rho proteins that freeze them into a GTP-bound state able to transduce signals. Our data strongly suggest that the activity of the Sec61 translocon, a gatekeeper of transmembrane protein synthesis, controls the activation of deamidated forms of Rho proteins. Indeed, we show that the inhibition of Sec61 translocon uncouples the deamidation of RhoA catalyzed by CNF1 from its GTP-loading and membrane anchoring. This suggests that activation of endogenous RhoA Q63E, generated by the intoxication of cells with CNF1, involves GEF factors that could modulate the effects of the toxin according to cell types and metabolic status. We provide evidence of a broad control operated by Sec61A1 on the activation of all three Rho proteins. The Rho GDP-dissociation inhibitor 1 (Rho GDI) is a shared regulator between Rac1, Cdc42, and RhoA that sequesters them as GDP-bound forms in the cytosol, thus protecting them against the UPS (53). Here, we found no detectable alteration of the prenylation of Rac1 in siSec61A1-treated cells when intoxicated by CNF1, thereby pointing to the possible interplay between Rho GDI and Sec61 translocon. Although this hypothesis is not supported by the absence of an increase in Rho GDI expression in cells, where the Sec61 translocon is inhibited, we cannot rule out the possible involvement of posttranslational modifications of Rho GDI in this process that would block the displacement of Rho from Rho GDI (54). Our results establish a hierarchical control of the activity of Rac1 GTPase by the Sec61 translocon, which points to a possible involvement of ER stress. Although both Tn and Tg induce ER stress, we show that only tunicamycin treatment blocks CNF1-induced depletion of Rac1. Thus, the control exerted by Sec61 translocon on Rac1 signaling most likely occurs independently of a general impact on ER stress.

We have developed a quantitative approach to measure the percentage of Rac1-positive cells in the whole cell population, allowing us to rank siRNAs as a function of the level of protection they confer against CNF1-mediated cellular depletion of Rac1. The reported lists of confirmed hits, including the 21 top-ranked hits and the best 22 hits having an annotated function in vesicular trafficking, provide information for future studies aimed at assessing the central role played by Rac1 and HACE1 in cell biology, cancer, and host-pathogen interactions. These data also pinpoint the importance of a group of factors involved in ER and Golgi trafficking and homeostasis, encompassing PITPNB, TMED9, SEL1L, SLC35E1, and GBF1 (Table 2). Whether these factors control the activation of Rac1 or its proteasomal degradation remains to be clarified. Nevertheless, we show that none of these hits had an impact on the deamidation of RhoA, e.g. able to impair CNF1 endocytosis and/or translocation to the cytosol. The shortlist of hits involved in vesicular trafficking also encompasses SNX4 (sorting nexin 4) and the small GTPase Rab11a, thereby pointing to the importance of vesicular recycling in CNF1-induced degradation of Rac1. Indeed, Rab11a can associate with HACE1 and is a known substrate of the E3 ligase HACE1 in the regulation of beta-adrenergic receptor recycling (28, 55). This raises the question of whether aberrant relocation of GTPase-impaired GTP-bound Rac1 Q61E, in recycling compartments, for example, accounts for its increased susceptibility to UPS. The list of confirmed hits also includes the component of endoplasmic reticulum-associated degradation (ERAD) SEL1L (suppressor/enhancer of Lin-12-like), which supports the dislocation of misfolded proteins from the luminal part of the ER to the cytosol for degradation. We cannot formally exclude the involvement of the ERAD machinery in CNF1-induced depletion of Rac1. Nevertheless, such regulation of Rac1 would involve a Sec61-independent pathway since the inhibition of Sec61 has no impact on ERAD (56). Alternatively, SEL1L may control Rac1 signaling in CNF1-treated cells via its reported influence on ER homeostasis and stress (57). Finally, we failed to identify regulatory components of the endolysosomal pathway in the list of best hits, except for Lu/BCAM. This points to technical limits in the screening method, although we had to exclude data from six plates that did not meet the quality control criteria. Thus, hits related to vesicular trafficking of CNF1 might be identified by performing this type of screen more than once. On the other hand, we found that the number of cells remaining after siRNA transfection, performed in the absence of serum in our study, had an impact on defining protection scores. This resulted in a counter-selection of siRNAs promoting cell rounding and detachment. Finally, in a similar screening method, we have previously isolated a small chemical inhibitor of the sorting of CNF1, which acts at the level of matured early endosomes (14). This raises the hypothesis that the absence of detection of siRNAs targeting components of the endolysosomal transport of CNF1 here reflects a redundancy in the activity of regulatory factors controlling the intracellular trafficking of the toxin.

Our data point to the importance of an underappreciated connection between protein biosynthesis in the ER and the regulation of Rac1 small GTPase.

## MATERIALS AND METHODS

### Cell culture, reagents, and antibodies

Primary human umbilical vein endothelial cells (HUVEC) up to six passages (PromoCell) were routinely cultured on gelatin 0.2% (Sigma) pre-coated dishes at 37°C, 5% CO_2_ in human endothelial SFM medium (Invitrogen) supplemented (SFMc) with 20% fetal bovine serum (FBS), 20 ng/mL fibroblast growth factor-2 (FGF-2) (PeproTech), 10 ng/mL epidermal growth factor (EGF) (PeproTech), and 1 mg/mL heparin (Sigma-Aldrich). MCF-12A mammary gland epithelial cells (CRL-10782, ATCC) were cultured in DMEM/F12 supplemented with 5% horse serum (Biowest), recombinant human epidermal growth factor (20 ng/mL) (Peprotech), human recombinant insulin (10  µg/mL) (Life Technologies), hydrocortisone (0.5  µg/mL) (Sigma-Aldrich), and cholera toxin (100 ng/mL) (Sigma-Aldrich). The primary and secondary screens were conducted with HUVEC at passage three in EGM-2 supplemented medium (Lonza) lacking VEGF and antibiotics, which otherwise interfere with the transfection efficiency. Recombinant CNF1 toxin was produced in *E. coli* and purified, as described in (58). Cells were intoxicated with CNF1 at the indicated time and concentrations in SFMc medium at 37°C, 5% CO_2_. The mycolactone was prepared from *Mycobacterium ulcerans* strain 1615 ATCC35840, as described in references 50, 59. Mycolactone was stored in DMSO at −20°C in amber glass tubes. The toxin was diluted extemporaneously in SFMc. Chemical inhibitors used in this study comprise 15 µM lovastatin (Enzo Life Sciences, #BML-G226), 2 µM thapsigargin (Enzo Life Sciences, #BML-PE180), and 2 µM tunicamycin (Tocris Bioscience, #3516). For immunoblots, the primary antibodies used were anti-GAPDH clone 411 (Santa Cruz, sc-47724, 1:5,000), anti-Rac1 clone 102 (BD Bioscience, #610651, 1:2,000), anti-RhoA clone 26c4 (Bertoglio laboratory, 1:2,500), anti-Cdc42 (Santa Cruz, SC-8401), anti-beta-1 integrin clone 18 /CD29 (BD Transduction Laboratories, #610468, 1:1000), anti-Rho GDI clone G-2 (Santa Cruz, sc-373724, 1:15000), and anti-ATF4 clone D4B8 (Cell Signaling, #11815, 1:1,000). The 4',6-diamidino-2-phenylindole (DAPI) was purchased from Sigma-Aldrich (#B9542).

### High content screening

In total, 384-well μclear black plates (Greiner Bio-One, #781091) were coated with fibronectin at 15 µg/mL (BD Bioscience, #356008) for 2 h at 37°C and dried overnight. Volumes of 250 nL of siRNA at 10 µM from siGENOME SMARTpool siRNA (Dharmacon, G-004675-E2 Lot 11157) or control siRNA (siCtrl) (Eurogentec, SR-CL000-005) and siHACE1 (Santa Cruz, sc-95301) were dispensed in a total of 66 plates using Echo550 Liquid Handler (Labcyte). The screen was performed in three runs of 22 plates each. A volume of 10 µL of an Opti-MEM Reduced Serum Medium (Gibco) containing 0.325 µL of HiPerfect transfection reagent (Qiagen) was added to each siRNA-containing well using a Microplate Washer Dispenser EL406 (Biotek). After 20 min at room temperature (RT), a volume of 40 µL of EGM-2 medium (Lonza) containing 2,000 HUVEC was added to each well using the same dispenser. Plates were then incubated at 37°C in 5% CO_2_ for 48 h. The next steps were conducted on the laboratory automation platform, Biocel System, operated with VWorks software (Agilent Technologies). The medium was removed before intoxicating cells with CNF1 at 1 nM in a volume of 50 µL of SFMc for 6 h. Cells were washed once with 50 µL of PBS before fixation in PBS containing 4% paraformaldehyde for 20 min at RT. Cells were washed in PBS and stained as follows. Briefly, cells were permeabilized in 50 µL of 0.5% Triton X-100 in PBS containing 0.1% BSA (Sigma) for 1 h. Each washing step was performed with 50 µL of PBS. Cells were incubated for 1 h with 30 µL of anti-Rac1 monoclonal antibodies (1:500). Cells were washed twice in PBS and incubated with secondary donkey anti-mouse antibodies coupled to AlexaFluor 488 (Alexa-488) (Invitrogen) (1:800) together with 1 µg/mL of 4',6-diamidino-2-phenylindole (DAPI). The plates were washed twice with PBS. Confocal images were taken using the automated confocal microscope IN Cell Analyzer 6000 (GE Healthcare) equipped with a dry objective 20×. Images of four fields were acquired for each well (*n* = 650 cells per well approximately). DAPI-stained nuclei were detected using 405 nm laser lines with a 450/50 nm emission filter. Endogenous Rac1 signal intensities were recorded using 488 nm laser lines with 540/75 nm emission filters. Images were analyzed by a multi-parameter approach using the Columbus image analysis software (Perkin Elmer, version 2.3.1) and the algorithm reported in Figure S4.

### Data formatting and normalization

We implemented a quantitative method of immunofluorescence signal processing to rank the protective effect of each siRNA on CNF1-induced depletion of Rac1. Cell segmentation was achieved based on the detection of DAPI signal intensities between nuclei (high intensity) and the cytoplasm (low intensity) using an algorithm implemented on Columbus software (Fig. S5). We next extracted quantitative information on Rac1 signal per cell corresponding to Alexa-488 signal intensities in segmented cell cytosol to define the number of Rac1-positive cells within the cell population. Thresholds set to define Rac1-positive cells for each siRNA correspond to mean (a.u. > 800) and minimum (a.u. > 300) Rac1 immunofluorescence intensities in siCtrl-transfected cells left untreated versus treated with CNF1. The quality control of the screen used the strictly standardized mean difference (SSMD) parameter based on positive (Posctrl, siCtrl, *n* = 10 wells) and negative (Negctrl, siCtrl + CNF1, *n* = 10 wells) controls per plate. In total, we removed from data analysis six plates with SSMD values >−5. Percentages of Rac1-positive cells defined in each condition were normalized plate by plate, giving a Rscreenorm score (Rscr), as described in (42). All siRNAs were ranked according to their values of Rscr, using the open-source R software (https://www.r-project.org):


$$R_{scr}=\frac{R_{SiRNAi}-median\;Neg\;{ctrl}_{plate\;i}}{median\;Pos\;{ctrl}_{plate\;i}-median\;Neg\;{ctrl}_{plate\;i}}$$

### Confirmatory screens

A total of 21 siRNAs from the list of top-ranked siRNA were re-ordered as a pool of 4 siRNAs (Dharmacon) and quantified for their efficacy against CNF1-mediated degradation of Rac1, as described in the primary screen. We have also short-listed 22 siRNAs targeting genes among the first 260 hits with scores above >0.6 and having an annotated function in vesicular trafficking. These siRNAs were re-ordered and screened for their efficacy against cellular depletion of Rac1 induced by CNF1, as described in the primary screen. For confirmatory screens, each siRNA confirmation was performed at least in triplicate in 384-well plates from three independent experiments.

### Transfection procedure

HUVECs were seeded at sub-confluence. The next day, transfection was carried out with 100 nM of siRNAs prepared in a mix of serum-free OptiMEM (Gibco) and PolyMag reagent (OZ Biosciences). The mix was incubated at RT for 20 min and then added to the cells. The dishes were then placed on a magnetic plate for 15 min at 37°C. After incubation time, the cells were left in the incubator for 1 h; then, the medium was exchanged for SFMc. Cells were next incubated at 37°C for 48 h. The list of the siRNAs used in this study is described in Table S2. The reverse transfection in 384-well plates used conditions of the high-throughput screen.

### Western blot analysis

Cells were lysed in 2× Laemmli buffer (Sigma-Aldrich) and boiled for 10 min at 100°C. Samples were resolved on 4–12% SDS-polyacrylamide gels and transferred to 0.45 µm PVDF-nitrocellulose membranes (GE Healthcare). Proper protein transfer was monitored with Ponceau S (Biorad) prior to blocking with 5% milk in Tris-buffer saline (TBS), Tween-20 at 0.005% (TBS-T) (Euromedex) for 1 h. Membranes were washed three times in TBS-T and incubated for 2 h with primary antibodies. Membranes were washed with TBS-T and incubated with anti-mouse or anti-rabbit horseradish peroxidase (HRP)-conjugated secondary antibodies (Dako) for 1 h. Signals were revealed using Immobilon Western Chemiluminescent HRP Substrate (Merck) on iBright CL1500 (Thermofisher). Protein signals were quantified using Image J software V2.3.1.

### Pulldown of active Rho GTPases

Pulldowns of the GTP-bound active form of Rho GTPases were conducted as described in (58). HUVECs treated with siRNA or pharmacological inhibitors were intoxicated with CNF1 toxin for 2 h. Dishes were washed in PBS and lysed in the Rac lysis buffer (25 mM HEPES, 150 mM NaCl, 5 mM MgCl_2_, 0.5% Triton X-100, 4% glycerol, 20 mM beta-glycerophosphate, and 10 mM NaF, pH 7.4) supplemented with Pierce EDTA-free protease inhibitors (Thermo Scientific, A32965). Cells were lysed through a 25G needle, and lysates were centrifuged at 12,000 *× g* for 10 min at 4°C to remove debris. Cell lysates were incubated with 30 µg of recombinant GST-PAK^70-106^ (46)-coated agarose beads for 40 min at 4°C. Beads were washed three times in Rac lysis buffer by centrifugation at 1,500 × *g*, 4°C for 5 min. Beads were resuspended in 50 µL of 1× Laemmli buffer. Proteins were resolved on a 4%–12% SDS-PAGE, and Rac1 was visualized by anti-Rac1 immunoblotting. The same protocol was followed to prepare cell lysates for active RhoA pulldown with the following modifications: cells were lysed in RhoA lysis buffer (50 mM Tris-HCl, 500 mM NaCl, 10 mM MgCl_2_, 1% Triton X-100, pH 7.4) freshly supplemented with 0.5% sodium deoxycholate, 0.1% SDS, and Pierce EDTA-free protease inhibitors. Cell lysates were incubated with 100 µg of recombinant GST-Rhotekin RBD-coated agarose beads (60).

### Membrane cytosol fractionation

A total of 4 × 10^6^ HUVECs were seeded in 100 mm plastic dishes the day before the experiment. Cells were scraped in 5 mL cold PBS, pelleted, and resuspended in 200 µL cold SI (250 mM sucrose, 3 mM imidazole, pH 7.4) buffer. Cells were lysed on ice by passing them 40 times through a 25G needle. Nuclei and cell debris were removed by centrifugation at 12,000 *× g* for 10 min at 4°C. The volume of each post-nuclear supernatant (PNS) was normalized if needed for ultracentrifugation at 100,000 × *g* for 1 h at 4°C. The supernatants were collected, while pellets corresponding to membrane fractions were homogenized in an equal volume of SI buffer. An equal volume of 20 µL of each fraction was resolved on SDS-PAGE and immunoblotted for Rac1, RhoA, Rho GDI, GAPDH, and beta-1 integrin.

### Triton X-114 phase separation

The prenylation of Rho GTPases was analyzed by Triton X-114 partition, as described in (45). Briefly, 3.5 × 10^6^ HUVEC were washed twice in PBS and detached in PBS EDTA 3 mM. After centrifugation, the cell pellet was resuspended in 0.4 mL PBS supplemented with 2% Triton X-114 buffer and incubated for 30 min at 4°C. Nuclei were removed by centrifugation at 16,000 × *g*, 4°C for 2 min. Supernatants were next incubated at 37°C for 10 min before centrifugation at 22,000 × *g* for 10 min at RT to allow efficient phase separation. The upper aqueous phase was collected. The lower detergent phase was washed with equal volumes of 0.1% Triton X-114 PBS lysis buffer in three cycles of phase separation, as described above. Both fractions were precipitated by adding methanol/chloroform/H_2_O (4:1:3). Air-dried pellets were then resuspended in 60 µL of 1× Laemmli buffer and protein resolved on 4–12% SDS-PAGE.

### RNA extraction and qRT-PCR

RNAs were extracted from HUVEC using the RNeasy Mini Kit (Qiagen, 74104) according to the manufacturer’s procedure. Reverse transcription was carried out with 1 µg of total RNA material and SuperScript IV Reverse Transcriptase (Invitrogen, #18090050). Quantitative PCR was performed with Master Mix PCR Power SYBR Green (Applied Biosystems, #4367659) and primers listed below. Conditions of qPCR were set as follows: 2 min at 50°C, 10 min at 95°C, 15 s at 95°C (40 cycles), and 1 min at 60°C on QuantStudio 7 Real-Time PCR (Thermo Fisher Scientific). The relative quantification was calculated by the 2^−ΔΔCt^ method with GAPDH as the housekeeping gene. Oligonucleotides used in this study were purchased from Sigma: for SEC61A1, 5′-GGACCGCTATCACCCTCTTTA and 5′-TCCATCAATGTGCCTCTGTTAGA; for GAPDH, 5′-AGGTGAAGGTCGGAGTCAACG and 5′- AGGGGTCATTGATGGCAACA.

### Statistical analysis

Statistical tests were performed using GraphPad Software Prism 9.2. All data are presented as the mean ± SEM. Detailed information on the number of replicates is provided in the figure legends. *P* values were calculated using one-way ANOVA with corrections for multiple comparisons or paired two-tailed *t*-test, as indicated in the figure legends. Significance levels are indicated as follows: not significant (ns) for *P* >  0.05, *P  ≤  0.05, **P  ≤  0.01, ***P  ≤  0.001, ****P  ≤  0.0001. Exact *P* values, when possible, are provided in the figure legends.

## Data Availability

The raw data set is available at https://doi.org/10.5281/zenodo.12626632.

## SUPPLEMENTAL MATERIAL

Figure S1 mbio.02585-24-s0001.tiff Screen of siRNAs interfering with CNF1-induced Rac1 depletion.

Figure S2 mbio.02585-24-s0002.tiff Cytosolic distribution of Rac1 signal upon Sec61A1 silencing.

Figure S3 mbio.02585-24-s0003.tiff Sec61 translocon is required for CNF1-mediated Cdc42 and RhoA activation.

Figure S4 mbio.02585-24-s0004.tiff Analyses of CNF1-induced deamidation of RhoA.

Figure S5 mbio.02585-24-s0005.tiff Workflow of the automated image analysis process.

Legends mbio.02585-24-s0006.docx Legends for supplemental files.

Supplemental Tables mbio.02585-24-s0007.pdf Tables S1 and S2.

ASM does not own the copyrights to Supplemental Material that may be linked to, or accessed through, an article. The authors have granted ASM a non-exclusive, world-wide license to publish the Supplemental Material files. Please contact the corresponding author directly for reuse.

## References

[1] Bement WM, Goryachev AB, Miller AL, von Dassow G. 2024. Patterning of the cell cortex by Rho GTPases. Nat Rev Mol Cell Biol 25:290–308. doi:10.1038/s41580-023-00682-z 38172611 PMC12706751

[2] Hodge RG, Ridley AJ. 2016. Regulating Rho GTPases and their regulators. Nat Rev Mol Cell Biol 17:496–510. doi:10.1038/nrm.2016.67 27301673

[3] Boquet P, Lemichez E. 2003. Bacterial virulence factors targeting Rho GTPases: parasitism or symbiosis? Trends Cell Biol 13:238–246. doi:10.1016/s0962-8924(03)00037-0 12742167

[4] Patel JC, Galán JE. 2005. Manipulation of the host actin cytoskeleton by Salmonella--all in the name of entry. Curr Opin Microbiol 8:10–15. doi:10.1016/j.mib.2004.09.001 15694851

[5] Aktories K, Barbieri JT. 2005. Bacterial cytotoxins: targeting eukaryotic switches. Nat Rev Microbiol 3:397–410. doi:10.1038/nrmicro1150 15821726

[6] Keestra AM, Bäumler AJ. 2014. Detection of enteric pathogens by the nodosome. Trends Immunol 35:123–130. doi:10.1016/j.it.2013.10.009 24268520 PMC3943588

[7] Mack NA, Whalley HJ, Castillo-Lluva S, Malliri A. 2011. The diverse roles of Rac signaling in tumorigenesis. Cell Cycle 10:1571–1581. doi:10.4161/cc.10.10.15612 21478669 PMC3127158

[8] Wittinghofer A, Vetter IR. 2011. Structure-function relationships of the G domain, a canonical switch motif. Annu Rev Biochem 80:943–971. doi:10.1146/annurev-biochem-062708-134043 21675921

[9] Tsoumtsa Meda LL, Landraud L, Petracchini S, Descorps-Declere S, Perthame E, Nahori MA, Ramirez Finn L, Ingersoll MA, Patiño-Navarrete R, Glaser P, Bonnet R, Dussurget O, et al.. 2022. The cnf1 gene is associated with an expanding Escherichia coli ST131 H30Rx/C2 subclade and confers a competitive advantage for gut colonization. Gut Microbes 14:2121577. doi:10.1080/19490976.2022.2121577 36154446 PMC9519008

[10] Kim KJ, Chung JW, Kim KS. 2005. 67-kDa laminin receptor promotes internalization of cytotoxic necrotizing factor 1-expressing Escherichia coli K1 into human brain microvascular endothelial cells. J Biol Chem 280:1360–1368. doi:10.1074/jbc.M410176200 15516338

[11] Piteau M, Papatheodorou P, Schwan C, Schlosser A, Aktories K, Schmidt G. 2014. Lu/BCAM adhesion glycoprotein is a receptor for Escherichia coli cytotoxic necrotizing factor 1 (CNF1). PLoS Pathog 10:e1003884. doi:10.1371/journal.ppat.1003884 24453976 PMC3894216

[12] Contamin S, Galmiche A, Doye A, Flatau G, Benmerah A, Boquet P. 2000. The p21 Rho-activating toxin cytotoxic necrotizing factor 1 is endocytosed by a clathrin-independent mechanism and enters the cytosol by an acidic-dependent membrane translocation step. Mol Biol Cell 11:1775–1787. doi:10.1091/mbc.11.5.1775 10793151 PMC14883

[13] Haywood EE, Ho M, Wilson BA. 2018. Modular domain swapping among the bacterial cytotoxic necrotizing factor (CNF) family for efficient cargo delivery into mammalian cells. J Biol Chem 293:3860–3870. doi:10.1074/jbc.RA117.001381 29371399 PMC5846154

[14] Wu Y, Mahtal N, Paillares E, Swistak L, Sagadiev S, Acharya M, Demeret C, Werf SVD, Guivel-Benhassine F, Schwartz O, et al.. 2022. C910 chemical compound inhibits the traffiking of several bacterial AB toxins with cross-protection against influenza virus. iScience 25:104537. doi:10.1016/j.isci.2022.104537 35769882 PMC9234246

[15] Pei S, Doye A, Boquet P. 2001. Mutation of specific acidic residues of the CNF1 T domain into lysine alters cell membrane translocation of the toxin. Mol Microbiol 41:1237–1247. doi:10.1046/j.1365-2958.2001.02596.x 11580831

[16] Schmidt G, Sehr P, Wilm M, Selzer J, Mann M, Aktories K. 1997. Gln 63 of Rho is deamidated by Escherichia coli cytotoxic necrotizing factor-1. Nature 387:725–729. doi:10.1038/42735 9192900

[17] Flatau G, Lemichez E, Gauthier M, Chardin P, Paris S, Fiorentini C, Boquet P. 1997. Toxin-induced activation of the G protein p21 Rho by deamidation of glutamine. Nature 387:729–733. doi:10.1038/42743 9192901

[18] Lerm M, Schmidt G, Goehring UM, Schirmer J, Aktories K. 1999. Identification of the region of Rho involved in substrate recognition by Escherichia coli cytotoxic necrotizing factor 1 (CNF1). J Biol Chem 274:28999–29004. doi:10.1074/jbc.274.41.28999 10506148

[19] Doye A, Mettouchi A, Bossis G, Clément R, Buisson-Touati C, Flatau G, Gagnoux L, Piechaczyk M, Boquet P, Lemichez E. 2002. CNF1 exploits the ubiquitin-proteasome machinery to restrict Rho GTPase activation for bacterial host cell invasion. Cell 111:553–564. doi:10.1016/s0092-8674(02)01132-7 12437928

[20] Torrino S, Visvikis O, Doye A, Boyer L, Stefani C, Munro P, Bertoglio J, Gacon G, Mettouchi A, Lemichez E. 2011. The E3 ubiquitin-ligase HACE1 catalyzes the ubiquitylation of active Rac1. Dev Cell 21:959–965. doi:10.1016/j.devcel.2011.08.015 22036506

[21] Daugaard M, Nitsch R, Razaghi B, McDonald L, Jarrar A, Torrino S, Castillo-Lluva S, Rotblat B, Li L, Malliri A, Lemichez E, Mettouchi A, Berman JN, Penninger JM, Sorensen PH. 2013. Hace1 controls ROS generation of vertebrate Rac1-dependent NADPH oxidase complexes. Nat Commun 4:2180. doi:10.1038/ncomms3180 23864022 PMC3759041

[22] Singh S, Machida S, Tulsian NK, Choong YK, Ng J, Shankar S, Liu Y, Chandiramani KV, Shi J, Sivaraman J. 2023. Structural basis for the enzymatic activity of the HACE1 HECT‐type E3 ligase through N‐terminal helix dimerization. Adv Sci (Weinh) 10. doi:10.1002/advs.202207672 PMC10520629 37537642

[23] Petracchini S, Hamaoui D, Doye A, Asnacios A, Fage F, Vitiello E, Balland M, Janel S, Lafont F, Gupta M, Ladoux B, Gilleron J, Maia TM, Impens F, Gagnoux-Palacios L, Daugaard M, Sorensen PH, Lemichez E, Mettouchi A. 2022. Optineurin links Hace1-dependent Rac ubiquitylation to integrin-mediated mechanotransduction to control bacterial invasion and cell division. Nat Commun 13:6059. doi:10.1038/s41467-022-33803-x 36229487 PMC9561704

[24] Goka ET, Lippman ME. 2015. Loss of the E3 ubiquitin ligase HACE1 results in enhanced Rac1 signaling contributing to breast cancer progression. Oncogene 34:5395–5405. doi:10.1038/onc.2014.468 25659579 PMC4633721

[25] Tortola L, Nitsch R, Bertrand MJM, Kogler M, Redouane Y, Kozieradzki I, Uribesalgo I, Fennell LM, Daugaard M, Klug H, et al.. 2016. The tumor suppressor hace1 is a critical regulator of TNFR1-mediated cell fate. Cell Rep 15:1481–1492. doi:10.1016/j.celrep.2016.04.032 27160902 PMC4893156

[26] Kogler M, Tortola L, Negri GL, Leopoldi A, El-Naggar AM, Mereiter S, Gomez-Diaz C, Nitsch R, Tortora D, Kavirayani AM, Gapp BV, Rao S, et al.. 2020. HACE1 prevents lung carcinogenesis via inhibition of RAC-family GTPases. Cancer Res 80:3009–3022. doi:10.1158/0008-5472.CAN-19-2270 32366477 PMC7611202

[27] Castillo-Lluva S, Tan C-T, Daugaard M, Sorensen PHB, Malliri A. 2013. The tumour suppressor HACE1 controls cell migration by regulating Rac1 degradation. Oncogene 32:1735–1742. doi:10.1038/onc.2012.189 22614015

[28] Tang D, Xiang Y, De Renzis S, Rink J, Zheng G, Zerial M, Wang Y. 2011. The ubiquitin ligase HACE1 regulates golgi membrane dynamics during the cell cycle. Nat Commun 2:501. doi:10.1038/ncomms1509 21988917 PMC3282116

[29] Huang S, Tang D, Wang Y. 2016. Monoubiquitination of syntaxin 5 regulates golgi membrane dynamics during the cell cycle. Dev Cell 38:73–85. doi:10.1016/j.devcel.2016.06.001 27404360 PMC4942811

[30] Phuyal S, Djaerff E, Le Roux AL, Baker MJ, Fankhauser D, Mahdizadeh SJ, Reiterer V, Parizadeh A, Felder E, Kahlhofer JC, Teis D, Kazanietz MG, Geley S, et al.. 2022. Mechanical strain stimulates COPII-dependent secretory trafficking via Rac1. EMBO J 41:e110596. doi:10.15252/embj.2022110596 35938214 PMC9475550

[31] Anitei M, Stange C, Parshina I, Baust T, Schenck A, Raposo G, Kirchhausen T, Hoflack B. 2010. Protein complexes containing CYFIP/Sra/PIR121 coordinate Arf1 and Rac1 signalling during clathrin-AP-1-coated carrier biogenesis at the TGN. Nat Cell Biol 12:330–340. doi:10.1038/ncb2034 20228810 PMC3241509

[32] Anitei M, Chenna R, Czupalla C, Esner M, Christ S, Lenhard S, Korn K, Meyenhofer F, Bickle M, Zerial M, Hoflack B. 2014. A high-throughput siRNA screen identifies genes that regulate mannose 6-phosphate receptor trafficking. J Cell Sci 127:5079–5092. doi:10.1242/jcs.159608 25278553

[33] Urra H, Henriquez DR, Cánovas J, Villarroel-Campos D, Carreras-Sureda A, Pulgar E, Molina E, Hazari YM, Limia CM, Alvarez-Rojas S, Figueroa R, Vidal RL, et al.. 2018. IRE1α governs cytoskeleton remodelling and cell migration through a direct interaction with filamin A. Nat Cell Biol 20:942–953. doi:10.1038/s41556-018-0141-0 30013108

[34] Voorhees RM, Hegde RS. 2016. Structure of the Sec61 channel opened by a signal sequence. Science 351:88–91. doi:10.1126/science.aad4992 26721998 PMC4700591

[35] Hall BS, Hill K, McKenna M, Ogbechi J, High S, Willis AE, Simmonds RE. 2014. The pathogenic mechanism of the Mycobacterium ulcerans virulence factor, mycolactone, depends on blockade of protein translocation into the ER. PLoS Pathog 10:e1004061. doi:10.1371/journal.ppat.1004061 24699819 PMC3974873

[36] Baron L, Paatero AO, Morel JD, Impens F, Guenin-Macé L, Saint-Auret S, Blanchard N, Dillmann R, Niang F, Pellegrini S, Taunton J, Paavilainen VO, et al.. 2016. Mycolactone subverts immunity by selectively blocking the Sec61 translocon. J Exp Med 213:2885–2896. doi:10.1084/jem.20160662 27821549 PMC5154940

[37] Demangel C. 2021. Immunity against Mycobacterium ulcerans: the subversive role of mycolactone. Immunol Rev 301:209–221. doi:10.1111/imr.12956 33607704

[38] Gérard SF, Hall BS, Zaki AM, Corfield KA, Mayerhofer PU, Costa C, Whelligan DK, Biggin PC, Simmonds RE, Higgins MK. 2020. Structure of the inhibited state of the Sec translocon. Mol Cell 79:406–415. doi:10.1016/j.molcel.2020.06.013 32692975 PMC7427319

[39] Itskanov S, Wang L, Junne T, Sherriff R, Xiao L, Blanchard N, Shi WQ, Forsyth C, Hoepfner D, Spiess M, Park E. 2023. A common mechanism of Sec61 translocon inhibition by small molecules. Nat Chem Biol 19:1063–1071. doi:10.1038/s41589-023-01337-y 37169959 PMC11458068

[40] Hsieh LT-H, Dos Santos SJ, Hall BS, Ogbechi J, Loglo AD, Salguero FJ, Ruf M-T, Pluschke G, Simmonds RE. 2022. Aberrant stromal tissue factor localisation and mycolactone-driven vascular dysfunction, exacerbated by IL-1β, are linked to fibrin formation in Buruli ulcer lesions. PLoS Pathog 18:e1010280. doi:10.1371/journal.ppat.1010280 35100311 PMC8846541

[41] Demangel C, High S. 2018. Sec61 blockade by mycolactone: a central mechanism in Buruli ulcer disease. Biol Cell 110:237–248. doi:10.1111/boc.201800030 30055020

[42] Bachas C, Hodzic J, van der Mijn JC, Stoepker C, Verheul HMW, Wolthuis RMF, Felley-Bosco E, van Wieringen WN, van Beusechem VW, Brakenhoff RH, de Menezes RX. 2018. Rscreenorm: normalization of CRISPR and siRNA screen data for more reproducible hit selection. BMC Bioinform 19:301. doi:10.1186/s12859-018-2306-z PMC6102854 30126372

[43] Hoffmann C, Aktories K, Schmidt G. 2007. Change in substrate specificity of cytotoxic necrotizing factor unmasks proteasome-independent down-regulation of constitutively active RhoA. J Biol Chem 282:10826–10832. doi:10.1074/jbc.M610451200 17296609

[44] Gil G, Faust JR, Chin DJ, Goldstein JL, Brown MS. 1985. Membrane-bound domain of HMG CoA reductase is required for sterol-enhanced degradation of the enzyme. Cell 41:249–258. doi:10.1016/0092-8674(85)90078-9 3995584

[45] Taguchi Y, Schätzl HM. 2014. Small-scale Triton X-114 extraction of hydrophobic proteins. Bio Protoc 4:e1139. doi:10.21769/BioProtoc.1139 PMC5697781 29170741

[46] Manser E, Loo TH, Koh CG, Zhao ZS, Chen XQ, Tan L, Tan I, Leung T, Lim L. 1998. PAK kinases are directly coupled to the PIX family of nucleotide exchange factors. Mol Cell 1:183–192. doi:10.1016/s1097-2765(00)80019-2 9659915

[47] Walter P, Ron D. 2011. The unfolded protein response: from stress pathway to homeostatic regulation. Science 334:1081–1086. doi:10.1126/science.1209038 22116877

[48] Morel J-D, Paatero AO, Wei J, Yewdell JW, Guenin-Macé L, Van Haver D, Impens F, Pietrosemoli N, Paavilainen VO, Demangel C. 2018. Proteomics reveals scope of mycolactone-mediated Sec61 blockade and distinctive stress signature. Mol Cell Proteomics 17:1750–1765. doi:10.1074/mcp.RA118.000824 29915147 PMC6126388

[49] Ogbechi J, Hall BS, Sbarrato T, Taunton J, Willis AE, Wek RC, Simmonds RE. 2018. Inhibition of Sec61-dependent translocation by mycolactone uncouples the integrated stress response from ER stress, driving cytotoxicity via translational activation of ATF4. Cell Death Dis 9:397. doi:10.1038/s41419-018-0427-y 29540678 PMC5852046

[50] Domenger A, Choisy C, Baron L, Mayau V, Perthame E, Deriano L, Arnulf B, Bories JC, Dadaglio G, Demangel C. 2022. The Sec61 translocon is a therapeutic vulnerability in multiple myeloma. EMBO Mol Med 14:e14740. doi:10.15252/emmm.202114740 35014767 PMC8899908

[51] Li WW, Alexandre S, Cao X, Lee AS. 1993. Transactivation of the grp78 promoter by Ca2+ depletion. A comparative analysis with A23187 and the endoplasmic reticulum Ca(2+)-ATPase inhibitor thapsigargin. J Biol Chem 268:12003–12009. 8505325

[52] Heifetz A, Keenan RW, Elbein AD. 1979. Mechanism of action of tunicamycin on the UDP-GlcNAc:dolichyl-phosphate Glc-NAc-1-phosphate transferase. Biochemistry 18:2186–2192. doi:10.1021/bi00578a008 444447

[53] Boulter E, Garcia-Mata R, Guilluy C, Dubash A, Rossi G, Brennwald PJ, Burridge K. 2010. Regulation of Rho GTPase crosstalk, degradation and activity by RhoGDI1. Nat Cell Biol 12:477–483. doi:10.1038/ncb2049 20400958 PMC2866742

[54] Kuhlmann N, Wroblowski S, Knyphausen P, de Boor S, Brenig J, Zienert AY, Meyer-Teschendorf K, Praefcke GJK, Nolte H, Krüger M, Schacherl M, Baumann U, James LC, Chin JW, Lammers M. 2016. Structural and mechanistic insights into the regulation of the fundamental Rho regulator RhoGDIα by lysine acetylation. J Biol Chem 291:5484–5499. doi:10.1074/jbc.M115.707091 26719334 PMC4786691

[55] Lachance V, Degrandmaison J, Marois S, Robitaille M, Génier S, Nadeau S, Angers S, Parent JL. 2014. Ubiquitylation and activation of a Rab GTPase is promoted by a β₂AR-HACE1 complex. J Cell Sci 127:111–123. doi:10.1242/jcs.132944 24190883

[56] Grotzke JE, Kozik P, Morel JD, Impens F, Pietrosemoli N, Cresswell P, Amigorena S, Demangel C. 2017. Sec61 blockade by mycolactone inhibits antigen cross-presentation independently of endosome-to-cytosol export. Proc Natl Acad Sci USA 114:E5910–E5919. doi:10.1073/pnas.1705242114 28679634 PMC5530691

[57] Sun S, Shi G, Han X, Francisco AB, Ji Y, Mendonça N, Liu X, Locasale JW, Simpson KW, Duhamel GE, Kersten S, et al.. 2014. Sel1L is indispensable for mammalian endoplasmic reticulum-associated degradation, endoplasmic reticulum homeostasis, and survival. Proc Natl Acad Sci USA 111:E582–91. doi:10.1073/pnas.1318114111 24453213 PMC3918815

[58] Doye A, Boyer L, Mettouchi A, Lemichez E. 2006. Ubiquitin-mediated proteasomal degradation of Rho proteins by the CNF1 toxin. Methods Enzymol 406:447–456. doi:10.1016/S0076-6879(06)06033-2 16472677

[59] Rifflet A, Demangel C, Guenin-Macé L. 2022. Mycolactone purification from M. ulcerans cultures and HPLC-based approaches for mycolactone quantification in biological samples. Methods Mol Biol 2387:117–130. doi:10.1007/978-1-0716-1779-3_13 34643908

[60] Ren XD, Kiosses WB, Schwartz MA. 1999. Regulation of the small GTP-binding protein Rho by cell adhesion and the cytoskeleton. EMBO J 18:578–585. doi:10.1093/emboj/18.3.578 9927417 PMC1171150

