# Supplementary figures and images for: Screening of gene function in cell intoxication by CNF1 links Sec61 translocon to Rac1 GTPase activity
Source: mBio. 2025 Oct 6;16(11):e02585-24. doi: 10.1128/mbio.02585-24 (PMC12607883; doi:10.1128/mbio.02585-24)

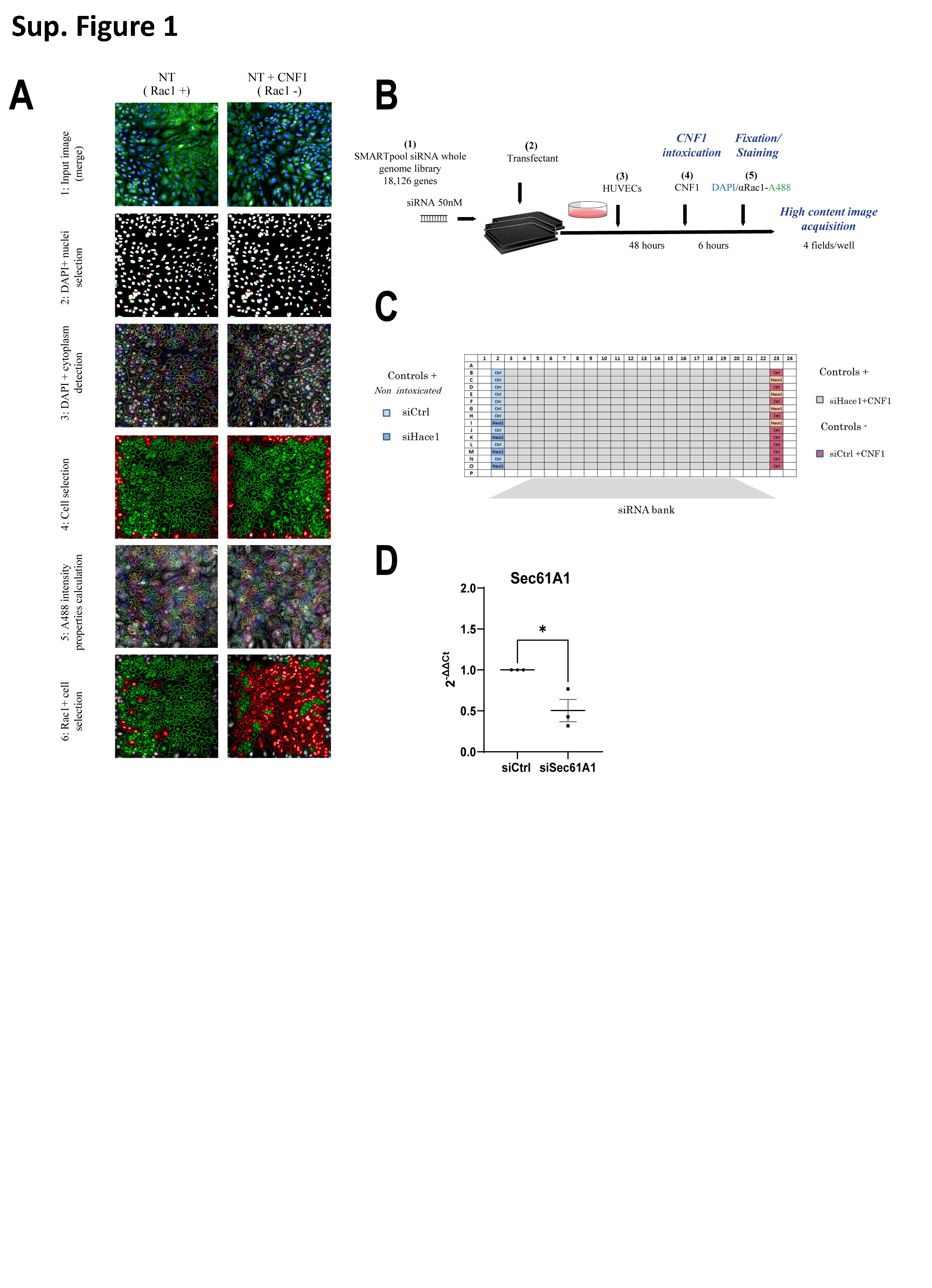

Supplement: Figure S1 — Screen of siRNAs interfering with CNF1-induced Rac1 depletion. [file mbio.02585-24-s0001.tiff]

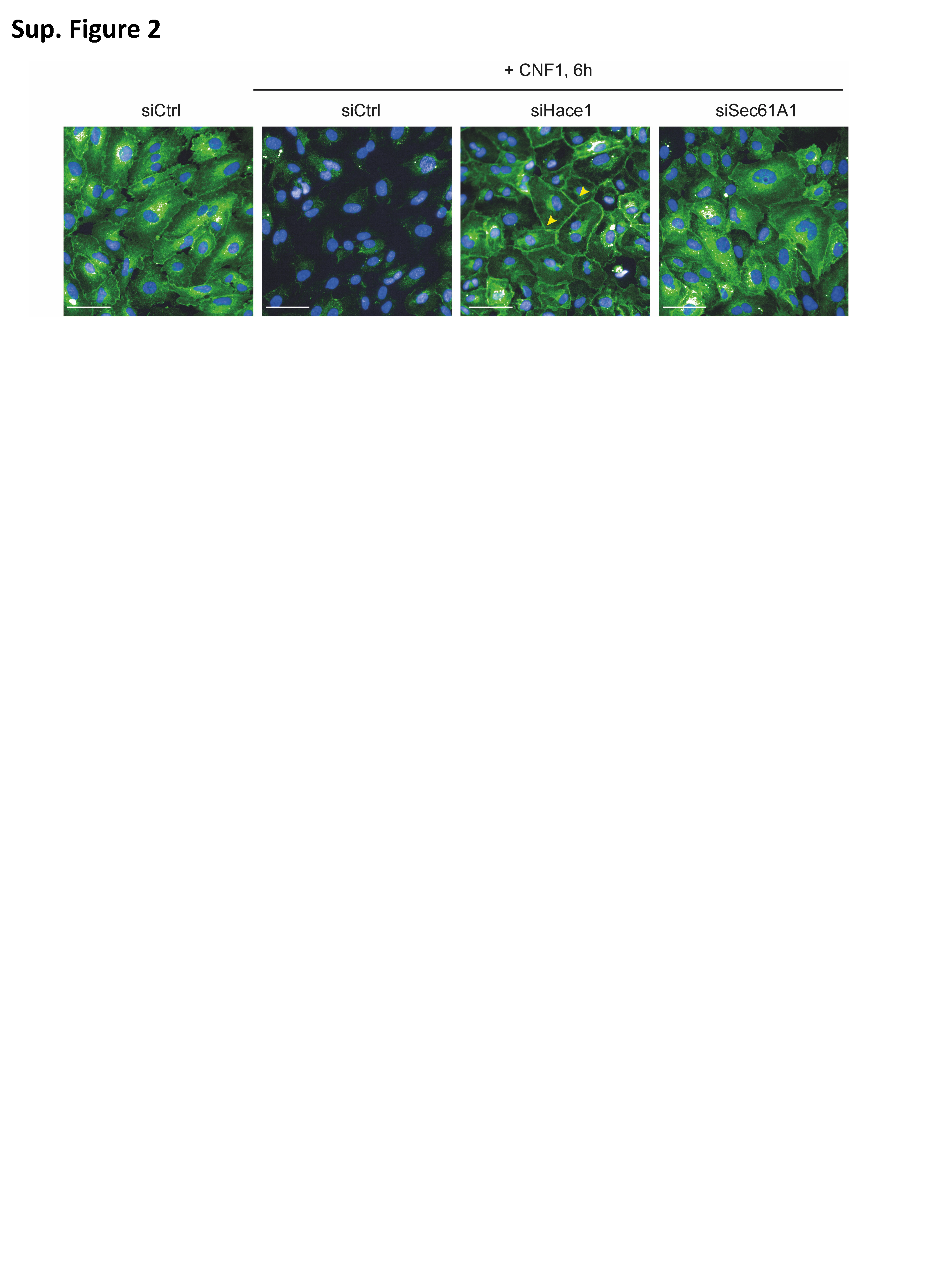

Supplement: Figure S2 — Cytosolic distribution of Rac1 signal upon Sec61A1 silencing. [file mbio.02585-24-s0002.tiff]

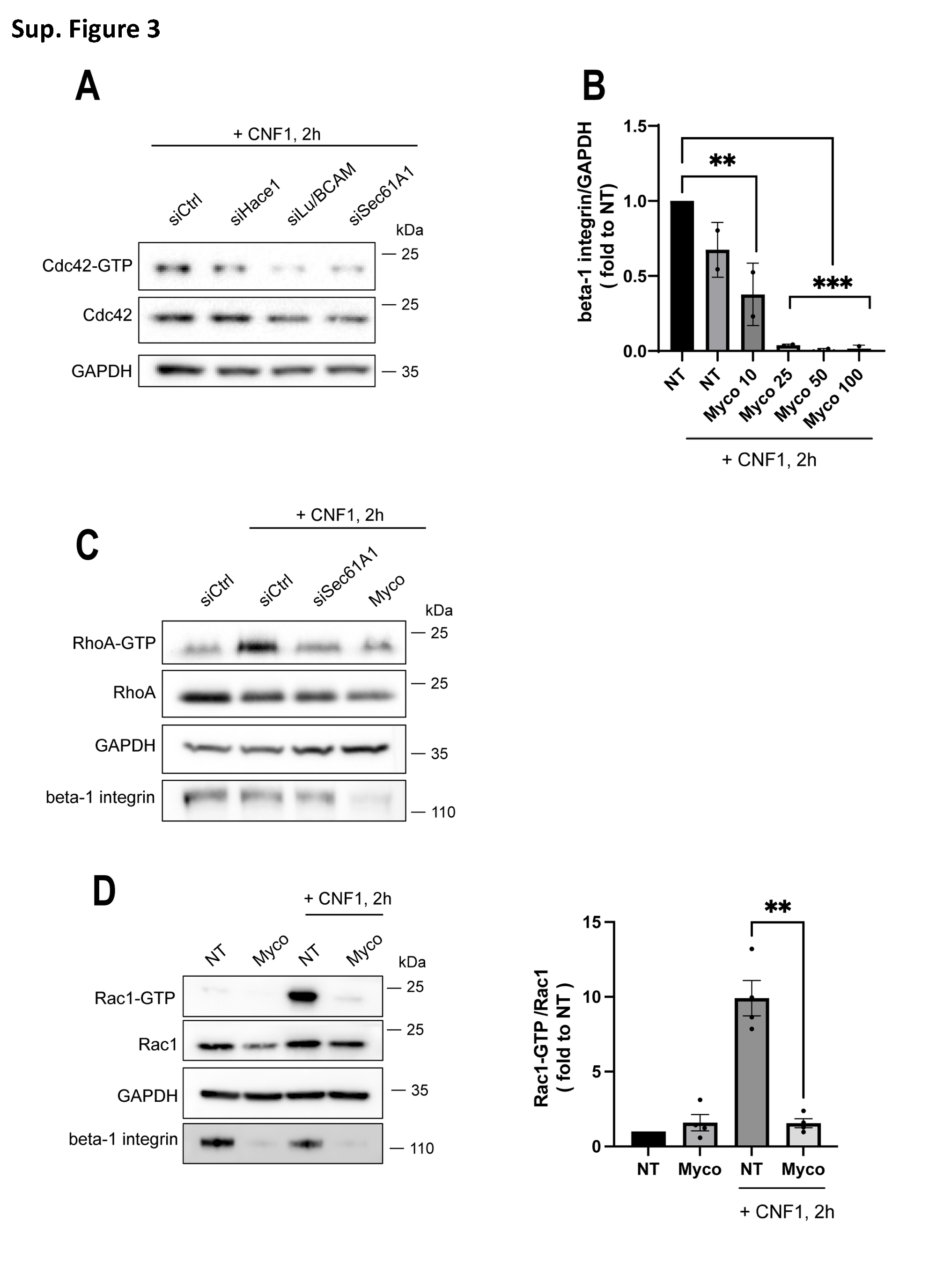

Supplement: Figure S3 — Sec61 translocon is required for CNF1-mediated Cdc42 and RhoA activation. [file mbio.02585-24-s0003.tiff]

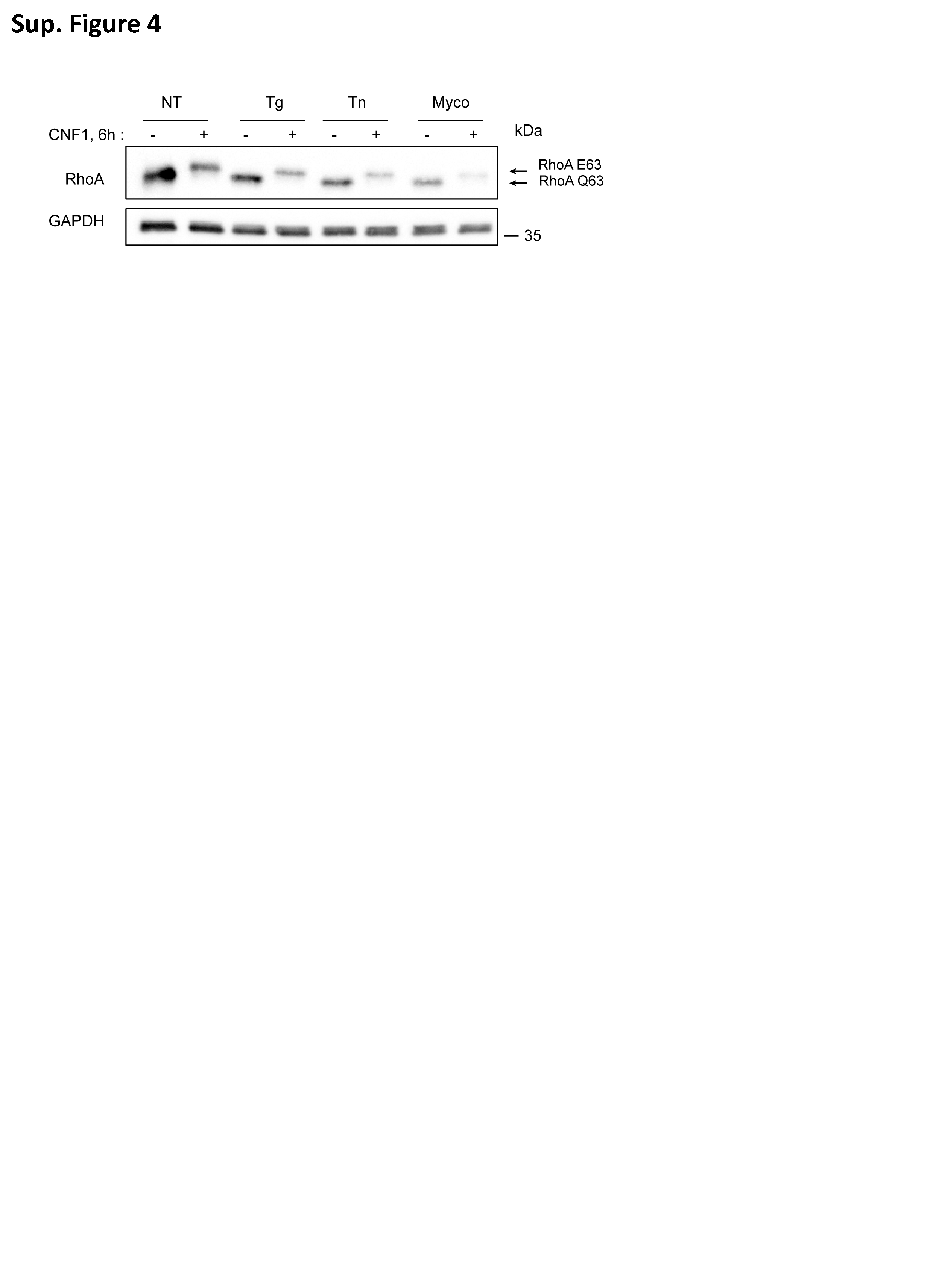

Supplement: Figure S4 — Analyses of CNF1-induced deamidation of RhoA. [file mbio.02585-24-s0004.tiff]

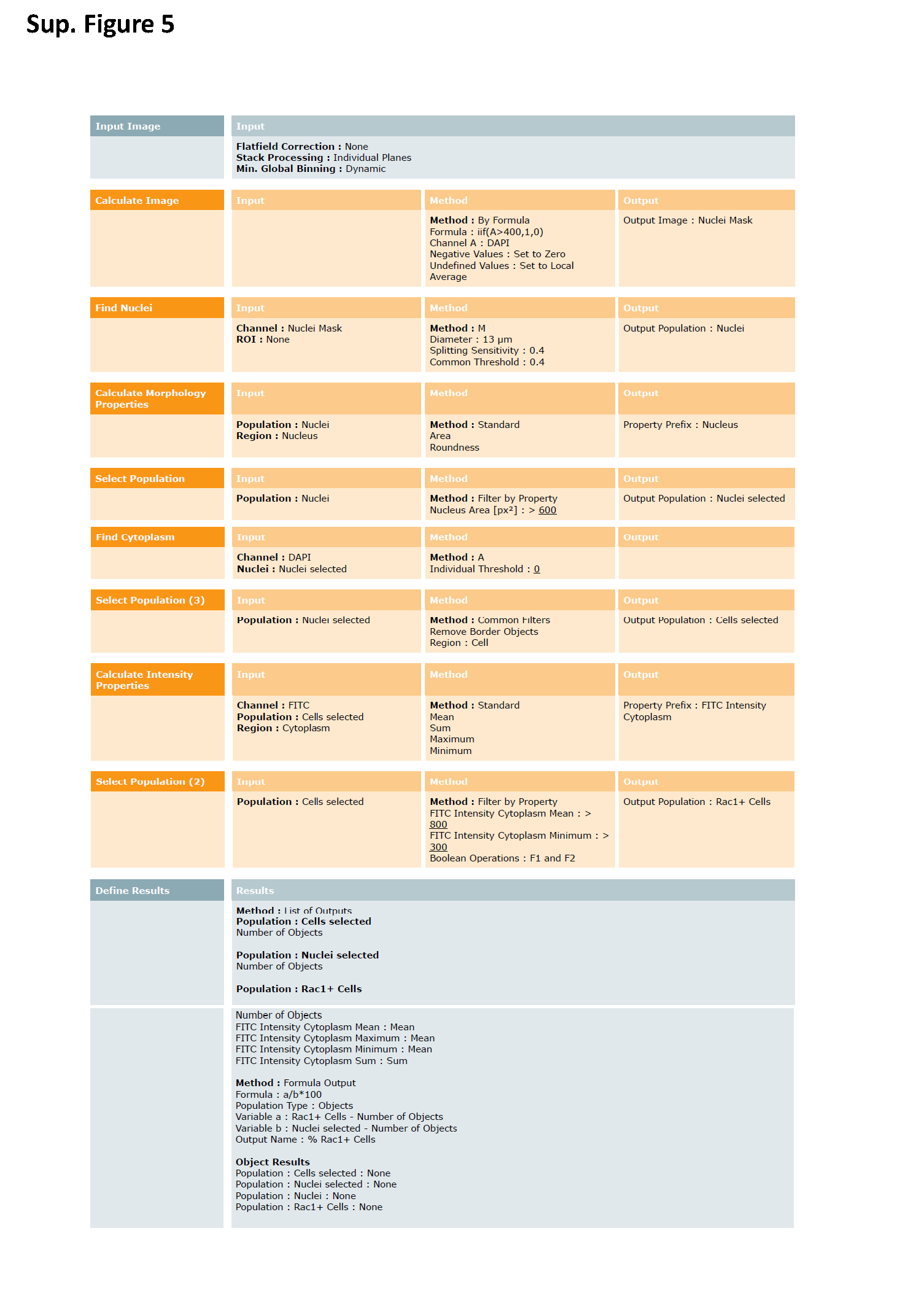

Supplement: Figure S5 — Workflow of the automated image analysis process. [file mbio.02585-24-s0005.tiff]
