# Supplementary material for: Screening of gene function in cell intoxication by CNF1 links Sec61 translocon to Rac1 GTPase activity
Source: mBio. 2025 Oct 6;16(11):e02585-24. doi: 10.1128/mbio.02585-24 (PMC12607883; doi:10.1128/mbio.02585-24)
Supplement: Legends — for supplemental files. [file mbio.02585-24-s0006.docx]

**SUPPLEMENTAL MATERIAL**

**SUPPLEMENTARY TABLE 1: top-ranked siRNA-targeted genes according to primary screen**

The table shows a list of the 260 first-ranked siRNAs from the primary screen. Columns from left to right: gene number, gene name (Ref_seq), Rscr value of Rac1-positive cells. Hace1 and Lu/BCAM are highlighted in pale green.

**SUPPLEMENTARY TABLE 2: list of siRNAs used in this study**

**SUPPLEMENTARY FIGURE 1: Screen of siRNAs interfering with CNF1-induced Rac1 depletion**

**A)** Image analysis workflow on Columbus software for the quantification of the number of Rac1-positive cells per condition. Nuclei and cell segmentation were based on the DAPI signal. Incomplete cells at the edge of the field were removed. The Alexa-488 signals (Rac1 signals) were quantified for each cell. Rac1-positive cells were defined according to the defined mean and minimum intensity thresholds of Alexa-488 signals set to 800 and 300, respectively. Cells were classified as positive (green) or negative (red) and the percentages of Rac1-positive cells were calculated per well.

**B)** Scheme illustrating the screening automated workflow on the Agilent Biocel platform. HUVEC were transfected with the whole human genome siRNA library SMARTpool encompassing 18,126 siRNA-targeting genes with HiPerfect transfectant for 48 hours. Cells were intoxicated with CNF1 at 1 nM for 6 hours before fixation and immunostaining with primary anti-Rac1 antibodies and secondary Alexa-488-cross-linked secondary antibodies. Cells were incubated with DAPI to stain nuclei. Images were acquired on an In cell 600 high-content microscope equipped with an objective 20X, 4 fields per well.

**C)** 384-well plate layout for the screen. Controls were added in columns 2 and 23 for each 384 well plate. The layout shows the position of CNF1-untreated siCtrl and siHace1 positive controls, CNF1-treated siHace1 positive controls as well as CNF1-treated siCtrl negative controls. All the siRNAs of the library (siRNA bank) were dispensed between control columns. All wells located on the edges were filled with medium to avoid liquid evaporation.

**D)** Graphs of relative fold-changes of mRNA levels (2^- ΔΔCt^) determined by RT-qPCR. HUVEC were transfected with siCtrl or siSec61A1 for 48 hours. Bars show mean ± sem. Each dot corresponds to an individual value from *n*= 3 independent experiments. Unpaired Student t test, two-tailed, Sec61A1: siCtrl vs siSec61A1, **P* = 0.0215

**SUPPLEMENTARY FIGURE 2: Cytosolic distribution of Rac1 signal upon Sec61A1 silencing**

Representative confocal images of endogenous Rac1 immunofluorescent signal (green) in HUVEC transfected with siCtrl, siHace1 or siSec61A1 and treated with CNF1 at 1 nM for 6 hours or left untreated. Arrowhead show accumulation of Rac1 signal at the periphery of cells. Confocal images were acquired on a spinning disk at the objective 20x. Nuclei were stained with DAPI (blue. Scale bar, 50 μm.

**SUPPLEMENTARY FIGURE 3: Sec61 translocon is required for CNF1-mediated Cdc42 and RhoA activation**

**A)** Immunoblots anti-Cdc42 shows levels of GTP-bound Cdc42 (Cdc42-GTP) associated with GST-PAK coated beads and total Cdc42 protein levels from cells transfected with siCtrl, siHace1, siLu/BCAM, or siSec61A1 prior to intoxication with CNF1 at 1 nM for 2 hours. Immunoblot anti-GAPDH shows relative protein levels engaged in the pulldown experiments. One representative experiment, *n*=2.

**B)** Graph shows levels of inhibition of beta-1 integrin expression after cell treatment with different concentrations of mycolactone ranging from 10 to 100 nM. Bars show mean ± sd. Each dot corresponds to an individual value from *n*=2 independent experiments. One WAY-ANOVA with Dunnett’s correction, NT vs Myco 10, ***P* < 0.0056; NT vs Myco 25, ****P* = 0.0006; NT vs Myco 50 and Myco 100 ****P* = 0.005.

**C)** Immunoblots anti-RhoA show levels of GTP-bound RhoA (RhoA-GTP) associated with GST-Rhotekin coated beads and relative cell levels of RhoA. Cells were transfected 48 hours with siCtrl, or siSec61A1 or treated 24 hours with 100 nM mycolactone. Cells were left untreated or treated with CNF1 at 1 nM for 2 hours. Immunoblot anti-beta-1 integrin shows the inhibition of beta-1 integrin expression. Immunoblot anti-GAPDH shows relative protein levels engaged in the pulldown experiments. One representative experiment, *n*=2.

**D)** Left panel: immunoblots anti-Rac1 shows levels of GTP-bound Rac1 (Rac1-GTP) associated with GST-PAK coated beads and total Rac1 protein levels (Rac1) in MCF12A cells treated 24 hours with 100 nM mycolactone and CNF1 at 1 nM for 2 hours, as indicated. Immunoblot anti-GAPDH shows relative protein levels engaged in the pulldown experiments. One representative experiment, *n*=4. Immunoblot anti-beta-1 integrin shows the inhibition of beta-1 integrin expression. Right graph: show the quantifications of Rac1-GTP signal normalized to total Rac1 (Rac1). Bars correspond to mean ± sem. Each dot corresponds to an individual value from *n*=4 independent experiments. Two-tailed student t-test: **P*=0.0049.

**SUPPLEMENTARY FIGURE 4: Analyses of CNF1-induced deamidation of RhoA**

Immunoblot anti-RhoA shows the electromobility shift of the deamidated form of RhoA (RhoA E63) in CNF1-intoxicated cells versus wildtype RhoA (RhoA Q63). Cells were not treated (NT) or treated with 2 µM of tapsigargin (Tg), 2 µM of tunicamycin (Tn) or 100nM mycolactone (Myco) for 24 hours prior to intoxication with 1 nM of CNF1 for 6 hours. Protein lysates were resolved on a 15% SDS-PAGE containing 1 M urea. Immunoblot GAPDH shows equal protein loading. One representative experiment from *n*=2.

**SUPPLEMENTARY FIGURE 5: Workflow of the automated image analysis process.**The multi-parametric dataset was generated using an image segmentation algorithm within the Columbus image analysis software (PerkinElmer, version 2.3.1).

**The complete dataset can be uploaded at** DOI 10.5281/zenodo.12188494
