# Supplementary material for: Screening of gene function in cell intoxication by CNF1 links Sec61 translocon to Rac1 GTPase activity
Source: mBio. 2025 Oct 6;16(11):e02585-24. doi: 10.1128/mbio.02585-24 (PMC12607883; doi:10.1128/mbio.02585-24)
Supplement: Supplemental Tables — Tables S1 and S2. [file mbio.02585-24-s0007.pdf]

Supplementary Table 1

N°	gene	Ref_Seq	Rscreenorm score
1	PTCD1	NM_015545	0,872911089
2	LIN9	NM_173083	0,872606874
3	HACE1	NM_020771	0,86795719
4	FLJ35119	NM_175871	0,860711811
5	ZNF598	NM_178167	0,859890806
6	HNRPA2B1	NM_002137	0,847962899
7	FBF1	NM_001080542	0,845174724
8	C22ORF4	NM_014346	0,837710515
9	HNRPUL2	NM_001079559	0,825368482
10	TMEM43	NM_024334	0,81369867
11	CYBASC3	NM_153611	0,813470768
12	SLC35E1	NM_024881	0,813237547
13	SEC61A1	NM_013336	0,808693992
14	VDP	NM_003715	0,808420017
15	LOC401190	NM_001029875	0,806966513
16	FLJ46082	NM_207417	0,801475942
17	LOC391123	NM_001013661	0,799062314
18	SH3D19	NM_001009555	0,798709419
19	OR2T6	NM_001005471	0,796810321
20	TRUB2	NM_015679	0,793671945
21	ANKRD32	NM_032290	0,792595474
22	LU	NM_001013257	0,788348389
23	SFPQ	NM_005066	0,787774139
24	OR8K5	NM_001004058	0,786923946
25	DKFZP566K0524	NM_001042365	0,786764976
26	PODXL	NM_005397	0,785810009
27	KHDRBS2	NM_152688	0,784931212
28	CX36	NM_020660	0,782436844
29	ZNF396	NM_145756	0,78162105
30	DDEF2	NM_003887	0,775204925
31	C9ORF37	NM_032937	0,773448485
32	DSG2	NM_001943	0,769379699
33	PHACTR1	NM_030948	0,768832825
34	TNFSF9	NM_003811	0,767856739
35	RP11-413M3,2	NM_001080849	0,767205
36	PRC1	NM_003981	0,765173925
37	HIPK2	NM_022740	0,764097756
38	LRCH1	NM_015116	0,761993181
39	PRB1	NM_005039	0,761539519
40	NDUFB10	NM_004548	0,759645465
41	TNFSF7	NM_001252	0,755238747
42	DKFZP762K222	NM_020225	0,75314833
43	IL12A	NM_000882	0,750283752
44	DVL1	NM_004421	0,749182885
45	PSMD13	NM_002817	0,748986872
46	KRTAP12-3	NM_198697	0,747260699
47	LOC389458	NM_203393	0,74390223
48	LOC151194	NM_145280	0,74109858
49	NS	NM_014366	0,738235861
50	ZBTB3	NM_024784	0,737171792
51	CSN2	NM_001891	0,735348424
52	CENTG2	NM_014914	0,734126769
53	KIAA1441	NM_020832	0,733749931
54	FLJ10613	NM_019067	0,732961835
55	C10ORF26	NM_001083913	0,731378964
56	EPPK1	NM_031308	0,727655911
57	KIAA1789	NM_001011657	0,726543182
58	CD48	NM_001778	0,724741959
59	FLJ38968	NM_152316	0,724549513
60	OR13J1	NM_001004487	0,724226516
61	RAPH1	NM_203365	0,722303763
62	COMMD5	NM_001081004	0,722064913
63	COAS2	NM_178230	0,721552513
64	CASC3	NM_007359	0,721466938
65	CRISPLD1	NM_031461	0,720905635
66	CGN	NM_020770	0,719970966
67	CENTD3	NM_022481	0,718201229
68	FLJ32356	NM_144671	0,717847846
69	EB-1	NM_020140	0,717664177
70	SEC63	NM_007214	0,714417389
71	SEL1L	NM_005065	0,713175188
72	PSD	NM_002779	0,712521567
73	C6ORF213	NM_001010852	0,709983936
74	NPIP	NM_006985	0,70972998
75	SPRY2	NM_005842	0,709283608
76	LOC345456	NM_001029886	0,70755804
77	CPSF6	NM_007007	0,707344066
78	FLJ20156	NM_017691	0,707251225
79	TESK1	NM_006285	0,706666753
80	SNRPA1	NM_003090	0,706483399
81	DEFB113	NM_001037729	0,705383779
82	GBF1	NM_004193	0,705177118
83	VTCN1	NM_024626	0,703792488
84	C14ORF45	NM_025057	0,703275188
85	FLJ34283	NM_182612	0,702408379
86	FLJ16636	NM_001001662	0,700172351
86	FLJ16636	NM_001001662	0,700172351
87	FRMD3	NM_174938	0,70014984
88	ZNF565	NM_152477	0,699683966
89	POP5	NM_015918	0,6992129
90	HIST1H1E	NM_005321	0,698596893
91	PSG1	NM_006905	0,697083274
92	C18ORF21	NM_031446	0,696148025
93	COTL1	NM_021149	0,693322742
94	DYRK4	NM_003845	0,692157222
95	MGC8974	NM_052940	0,691936481
96	LOC57146	NM_020422	0,691413691
97	LOC390637	NM_001013657	0,690748324
98	DKFZP761L1518	NM_198075	0,690665458
99	DCTN4	NM_016221	0,689070094
100	COMMD2	NM_016094	0,68831161
101	FLJ22624	NM_024808	0,687812735
102	MAP4K5	NM_006575	0,687560186
103	RDH10	NM_172037	0,687415091
104	PITPNB	NM_012399	0,687194246
105	OTOA	NM_170664	0,687099823
106	DKFZP434I1610	NM_144566	0,686985881
107	C10ORF99	NM_207373	0,686930571
108	ENG	NM_000118	0,685931772
109	SNTG2	NM_018968	0,685568072
110	CGI-121	NM_016058	0,684822724
111	FLJ12057	NM_024768	0,684612722
112	SPOP	NM_001007228	0,683466648
113	PTPN20A	NM_001042397	0,681970903
114	DKFZP434F0318	NM_030817	0,680314872
115	FLJ10634	NM_018163	0,677661767
116	MGC15716	NM_032370	0,677645568
117	FLJ10379	NM_018079	0,676343153
118	UBL3	NM_007106	0,67575821
119	EXOC7	NM_015219	0,675555458
120	FLJ36198	NM_173801	0,673694126
121	MGC26856	NM_152779	0,673172006
122	LOC51249	NM_016486	0,670598337
123	LYPD2	NM_205545	0,670214843
124	CNOT10	NM_015442	0,669842875
125	PX19	NM_013237	0,667830289
126	GTF3C5	NM_012087	0,666523879
127	DKFZP564O1664	NM_030800	0,666086307
128	MGC20533	NM_001011699	0,666075778
129	FLJ12969	NM_022838	0,666059404
130	FLJ31121	NM_144723	0,664482119
131	ORC4L	NM_002552	0,664188784
132	GDF6	NM_001001557	0,663836963
133	OPA3	NM_001017989	0,663045006
134	ZNF415	NM_018355	0,661986576
135	HIST1H2BI	NM_003525	0,660839318
136	UAP1	NM_003115	0,660047862
137	UMOD	NM_001008389	0,658954409
138	PB1	NM_181042	0,657034393
139	KIAA1862	NM_032534	0,656306385
140	DNAJC12	NM_021800	0,656293887
141	EMX1	NM_004097	0,655259216
142	MGC48998	NM_178550	0,652634538
143	COMMD8	NM_017845	0,65291238
144	LOC285382	NM_001025266	0,65203083
145	APRG1	NM_178339	0,651356128
146	ZFP64	NM_199427	0,650066228
147	AXIN2	NM_004655	0,649829089
148	HGS	NM_004712	0,649113669
149	GAP43	NM_002045	0,649042853
150	ZCCHC5	NM_152694	0,648041729
151	COP	NM_001017534	0,647829341
152	FLJ35725	NM_152544	0,647714367
153	C9ORF4	NM_014334	0,645481017
154	PSMD5	NM_005047	0,644076848
155	DPPA4	NM_018189	0,644049817
156	RAB41	NM_001032726	0,644047702
157	SLC2A7	NM_207420	0,643404859
158	KIAA0992	NM_016081	0,643310465
159	FLJ35630	NM_152618	0,643101207
160	HT2R55	NM_181429	0,642777191
161	FLJ10900	NM_018264	0,641902904
162	LARP	NM_015315	0,641684605
163	GIMAP6	NM_001007224	0,640621291
164	LOC646424	NM_001080525	0,639138354
165	FLJ90013	NM_153365	0,639090206
166	C17ORF38	NM_001010855	0,63866629
167	PRDM5	NM_018699	0,638623388
168	STK16	NM_001008910	0,638584219
169	FLJ10116	NM_018000	0,637650345
170	UTX	NM_021140	0,635802318
171	AQP2	NM_000486	0,635600058
172	STAG2	NM_006603	0,635339141
173	TTYH2	NM_052869	0,635031014
174	LOC152485	NM_178835	0,634354447
175	G0S2	NM_015714	0,634339307
176	LOC93349	NM_138402	0,633461267
177	ATP6V1F	NM_004231	0,633147683
178	KIAA1143	NM_020696	0,633108362
179	HT017	NM_020678	0,632343181
180	PSPHL	XM_926720	0,631876152
181	SNX4	NM_003794	0,631465068
182	MDM1	NM_020128	0,631255514
183	C9ORF13	NM_153366	0,630874138
184	OR51Q1	NM_001004757	0,630579708
185	VHLL	NM_001004319	0,630105676
186	FLJ45530	NM_001001682	0,629862118
187	IZUMO4	NM_001031735	0,629846527
188	RPH3AL	NM_006987	0,629138014
189	KRTAP23-1	NM_181624	0,629135989
190	PARG1	NM_004815	0,628900178
191	MGC39581	NM_152784	0,628878168
192	PFS2	NM_016095	0,628876632
193	SNRPD2	NM_004597	0,628364932
194	C14ORF108	NM_018229	0,627485007
195	TCF21	NM_003206	0,627017809
196	TMED9	NM_017510	0,626693311
197	SLC39A9	NM_018375	0,626093178
198	STARD3NL	NM_032016	0,625890383
199	XG	NM_175569	0,625265788
200	NYD-SP14	NM_031956	0,625025941
201	THSD6	NM_213604	0,624791173
202	CHCHD7	NM_001011671	0,624251777
203	LOC400566	NM_001013672	0,624206148
204	LOC127262	NM_182752	0,62385452
205	FAM18B	NM_016078	0,623738451
206	PVALB	NM_002854	0,623186041
207	MFAP4	NM_002404	0,6230571
208	MGC51082	NM_182498	0,622418915
209	MT1X	NM_005952	0,62193777
210	LOC390999	NM_001080830	0,621446785
211	STIP1	NM_006819	0,620260565
212	ARV1	NM_022786	0,61957413
213	LOC144983	NM_001011725	0,619396683
214	TAS2R10	NM_023921	0,619267733
215	C14ORF11	NM_018453	0,618649389
216	PCDHGB3	NM_018924	0,618495676
217	PP1665	NM_030792	0,618379972
218	ST18	NM_014682	0,61793887
219	DUSP15	NM_177991	0,617362188
220	ZNF208	NM_007153	0,616612124
221	ZNF286	NM_020652	0,616509368
222	MGC34648	NM_152660	0,616384956
223	OR8H3	NM_001005201	0,616038319
224	EI24	NM_001007277	0,616012699
225	AE2	NM_032264	0,615628081
226	LOC219527	NM_001005210	0,615519493
227	RDBP	NM_002904	0,615400464
228	FLJ32771	NM_145017	0,614568088
229	AKR7A3	NM_012067	0,61441419
230	SPATA7	NM_001040428	0,614256001
231	FLJ31882	NM_152460	0,614146883
232	LOC497661	NM_001035005	0,613852203
233	SELS	NM_018445	0,612553679
234	HIST1H4L	NM_003546	0,611975309
235	LOC199675	NM_174918	0,611912731
236	KIAA1946	NM_177454	0,6101752
237	LOC390980	NM_001023563	0,608972763
238	ULBP3	NM_024518	0,60828299
239	RAB11A	NM_004663	0,607978363
240	ELAVL2	NM_004432	0,607573167
241	FGF4	NM_002007	0,606973774
242	TLR2	NM_003264	0,606523463
243	ITGAL	NM_002209	0,606488423
244	FLJ10846	NM_018241	0,606466766
245	CRTAP	NM_006371	0,606264276
246	FLJ13841	NM_024702	0,605506889
247	IGSF4D	NM_153184	0,604753617
248	SPRY3	NM_005840	0,604159401
249	PFDN4	NM_002623	0,604100735
250	COL17A1	NM_000494	0,603299881
251	CDH11	NM_001797	0,603019126
252	LOC92906	NM_138394	0,603010925
253	JPH1	NM_020647	0,6026387
254	FLJ25078	NM_152485	0,60231356
255	OR4X2	NM_001004727	0,601426271
256	LOC157697	NM_207332	0,600763601
257	KIAA0377	NM_014659	0,600646912
258	FLJ10803	NM_018224	0,600601917
259	ZNF137	NM_003438	0,600035534

Sup. Table 2

Gene Symbol	GENE ID	Fournisseur	Catalog Number	Infos
Scramble	-	Eurogentec	SR-CL000-005	Nontargeting siRNA
HACE1	57531	SantaCruz	sc-95301	pool 3 siRNA
LU	4059	Dharmacon	L-010608-00	onTARGET smart pool
SEC61A1	29927	Dharmacon	L-021503-01	siGENOME smartpool
SEC61A2	55176	Dharmacon	L-013628-01	siGENOME smartpool
SEC61G	23480	Dharmacon	L-012509-01	siGENOME smartpool
SEC61B	10952	Dharmacon	L-021504-01	siGENOME smartpool
SEC62	7095	Dharmacon	L-010218-01	siGENOME smartpool
SEC63	11231	Dharmacon	L-004886-01	siGENOME smartpool
PTCD1	26024	Dharmacon	L-021366-01	siGENOME smartpool
LIN9	286826	Dharmacon	L-018918-01	siGENOME smartpool
SWSAP1	126074	Dharmacon	L-017913-01	siGENOME smartpool
ZNF598	90850	Dharmacon	L-007104-00	siGENOME smartpool
HNRNPA2B1	3181	Dharmacon	L-011690-01	siGENOME smartpool
TBC1D22A	25771	Dharmacon	L-009319-01	siGENOME smartpool
HNRNPUL2	221092	Dharmacon	L-032789-01	siGENOME smartpool
TMEM43	79188	Dharmacon	L-014342-02	siGENOME smartpool
CYB561A3	220002	Dharmacon	L-016374-02	siGENOME smartpool
RGS7BP	401190	Dharmacon	L-028495-02	siGENOME smartpool
CFAP77	389799	Dharmacon	L-032095-02	siGENOME smartpool
VSIG8	391123	Dharmacon	L-027730-02	siGENOME smartpool
SH3D19	152503	Dharmacon	L-024058-00	siGENOME smartpool
OR2T6	254879	Dharmacon	L-032462-02	siGENOME smartpool
TRUB2	26995	Dharmacon	L-013879-02	siGENOME smartpool
SLF1	84250	Dharmacon	L-014837-02	siGENOME smartpool
PTPN20	26095	Dharmacon	L-013909-00	siGENOME smartpool
SFPQ	6421	Dharmacon	L-006455-00	siGENOME smartpool
OR8K5	219453	Dharmacon	L-026626-02	siGENOME smartpool
KHDRBS2	202559	Dharmacon	L-016897-01	siGENOME smartpool
GJD2	57369	Dharmacon	L-020726-01	siGENOME smartpool
ZNF396	252884	Dharmacon	L-021304-02	siGENOME smartpool
PHACTR1	221692	Dharmacon	L-025063-02	siGENOME smartpool
LRCH1	23143	Dharmacon	L-014112-02	siGENOME smartpool
ASAP2	8853	Dharmacon	L-011544-00	siGENOME smartpool
ZNF687	57592	Dharmacon	L-007036-01	siGENOME smartpool
SEL1L	6400	Dharmacon	L-004885-00	siGENOME smartpool
AGAP1	116987	Dharmacon	L-020452-01	siGENOME smartpool
ARAP3	64411	Dharmacon	L-007052-00	siGENOME smartpool
ARF1	375	Dharmacon	L-011580-00	siGENOME smartpool
COMMD2	51122	Dharmacon	L-021024-02	siGENOME smartpool
COMMD5	28991	Dharmacon	L-015390-02	siGENOME smartpool
COMMD8	54951	Dharmacon	L-018218-02	siGENOME smartpool
CYB561A3	220002	Dharmacon	L-016374-02	siGENOME smartpool
EXOC7	23265	Dharmacon	L-021448-00	siGENOME smartpool
FAM109A	144717	Dharmacon	L-015976-01	siGENOME smartpool
GBF1	8729	Dharmacon	L-019783-00	siGENOME smartpool
GNL3	26354	Dharmacon	L-016319-01	siGENOME smartpool
HGS	9146	Dharmacon	L-016835-00	siGENOME smartpool
HIPK2	28996	Dharmacon	L-003266-00	siGENOME smartpool
PITPNB	23760	Dharmacon	L-006459-01	siGENOME smartpool
PODXL	5420	Dharmacon	L-010617-00	siGENOME smartpool
RAB11A	8766	Dharmacon	L-004726-00	siGENOME smartpool
RAB41	347517	Dharmacon	L-031575-01	siGENOME smartpool
SNX4	8723	Dharmacon	L-011520-00	siGENOME smartpool
TBC1D22A	25771	Dharmacon	L-009319-01	siGENOME smartpool
TMED9	54732	Dharmacon	L-007924-02	siGENOME smartpool
USO1	8615	Dharmacon	L-011501-01	siGENOME smartpool
